# Effect of photobiomodulation therapy on orthodontic-induced inflammatory root resorption in male albino rats

**DOI:** 10.1186/s12903-025-07217-2

**Published:** 2025-12-11

**Authors:** Ahmed M. Yassin, Afaf A. El-Sawa, Amira A. Elnozahy, Hagar S. Abdel Fattah

**Affiliations:** 1 Oral Biology Department, Faculty of Dentistry, Alexandria University, Champolion St. Azarita, Alexandria, 21521 Egypt; 2 Oral Biology Department, Faculty of Dentistry, Beirut Arab University, Beirut, Lebanon

**Keywords:** Photo-biomodulation therapy, Low-level laser therapy, Root resorption, Phototherapy, Orthodontic force magnitudes

## Abstract

**Background:**

This study aimed to assess histologically and histomorphometrically the effect of Photo-biomodulation therapy (PBM) on orthodontic-induced inflammatory root resorption (OIIRR) under relatively light- and heavy- orthodontic forces in rats.

**Methods:**

Thirty-two male Albino rats weighing between (250–300 g) were randomly assigned to four equal groups (gp) of 8 rats each. Control low-force gp (CL): The maxillary left 1st molar (M1) was subjected to 10–15 g orthodontic force by nickel titanium coil spring stretched between the M1 and maxillary incisors. Control high-force gp (CH): The M1 had heavier orthodontic force of 50 g. Laser low-force gp (LL): The M1 had orthodontic force of 10–15 g & PBM using GaAlAs diode laser (λ = 980 nm, 100 mW output power) at two points palatal and two buccal, with a continuous non-contact irradiating mode for 12 s for each point (energy of 1.2 J/point, fluence of 16.9 J/cm^2^). Laser high-force gp (LH): M1 had orthodontic force of 50 g & PBM. The rats were euthanized after 7- and 21-days intervals in each gp and the maxillae were dissected out, then serial transverse sections of the M1 were taken for histological and histomorphometric evaluation.

**Results:**

Our results showed significant decrease in the amount of OIIRR in the laser gps at day 21 in all force subgroups, without significant change at day 7.

**Conclusion:**

PBM can significantly reduce the overall amount of root resorption and enhance/accelerate the healing of OIIRR.

**Supplementary Information:**

The online version contains supplementary material available at 10.1186/s12903-025-07217-2.

## Highlights

• Photo-biomodulation (PBM) reduces the amount of orthodontic root resorption.

• PBM accelerates the tissue events during the orthodontic tooth movement (OTM).

• PMB improves the periodontal tissue blood supply and its related tissue events.

• PBM enhances regeneration and reorganization of the periodontal tissues after OTM.

**Supplementary Information:**

The online version contains supplementary material available at 10.1186/s12903-025-07217-2.

## Background

It has been reported that orthodontic-induced inflammatory root resorption (OIIRR) is the most frequent adverse consequences of orthodontic therapy [1]. OIIRR is thought to be a secondary consequence of the cellular activity related to the elimination of the hyaline zone at a periodontal ligament (PDL) that has been over-compressed [2]. 

Numerous etiological factors, either patient-specific or treatment mechanics-specific, had been hypothesised to affect the root resorption activity. These include genetic factors[3], age[4], force magnitude[5], treatment duration[6], tooth movement distance[7], tooth movement direction and force application type (continuous versus intermittent) [2]. 

Among the previously mentioned factors influencing the OIIRR, it has been suggested that the force magnitude factor has a crucial effect on the degree of root resorption [5, 8]. Ideal orthodontic tooth movement (OTM) produces the maximum tooth movement rate with the least risk of tissue damage and patient discomfort [9]. 

Various chemical and pharmacological agents, as well as surgical and nonsurgical procedures, have been studied in an effort to overcome and treat this issue. However, the majority of these treatment modalities are not clinically appropriate since they might have possible adverse consequences on OTM and the patient’s overall health and comfort. Photo-biomodulation therapy (PBM) does not produce any general adverse effects, discomfort, or impact the overall wellness state of the patients, unlike the majority of the alternative therapy approaches and on contrary to injectable drugs and orally-administered medications [10]. 

PBM is a photon therapy employs non-ionizing forms of light from sources such as lasers, LEDs, and broadband light in the visible and near-infrared spectrum to elicit biological alterations and therapeutic effects [11]. 

As OTM incorporates a coordinated inflammatory reaction, several studies had examined the impact of PBM on pain management[12], rate of OTM[13], bone remodeling [14] and root injury during OTM [15]. 

Due to the reparative, anti-inflammatory, and biostimulative properties of PBM, as well as the influence of laser irradiation on the osteoclastic and osteoblastic functions, several researches have been done to determine the effect of PBM on OIIRR with considerable controversy in the results [16–18]. 

To the best of what we know, all the previous studies in the scientific field, concerning the impact of PBM on OIIRR, evaluated the photo-biomodulation effect on the OIIRR using only one fixed orthodontic force magnitude in each study. Therefore, our study aimed to assess the impact of PBM on OIIRR using two different orthodontic force magnitudes in a rodent model. The null hypothesis of this experimental study was that there is no effect of PBM on OIIRR under different orthodontic force magnitudes. The alternative hypothesis was that the PBM could have effect on OIIRR under different orthodontic force magnitudes.

## Materials and methods

### Ethical approval

This research was conducted in accordance with the highest standards of ARRIVE guidelines approved by the Research Ethics Committee of the Faculty of Dentistry, Alexandria University for the conduct of research on experimental animals (Ethics Committee No:0200 − 12/2020 - IORG 0008839).

### Sample size Estimation

By applying a power of 80% to detect a standardized effect size in area of root resorption, and level of significance 95% (α = 0.05), the minimum sample size needed was 8 rats per group (four per time interval) with total sample size of 32 rats, based on previous studies [19, 20]. This animal number has been estimated according to specific calculations done in the Department of Medical Statistics Medical Research Institute, Alexandria University. The sample size was figured out utilizing G*Power software version 3.1.9.2[21].

### Experimental animals

Thirty-two 12-weeks-old healthy male Albino rats that weighed between (250–300 g) were involved in this study. The experimental animals were obtained and reared at the animal house of Medical Research Institute, Alexandria University. The animals were housed in collective smooth walled metallic cages, 4 rats per cage. The temperature was kept between 22 and 25 degrees Celsius, there was sufficient ventilation, as well as conventional light/dark cycle (12/12 h). the animals were able to consume food and water as much as they wanted. On every day throughout the trial, the standard dietary regimen was routinely replaced.

### Intervention

#### Grouping

The 32 rats (*n* = 32) were divided randomly and evenly into four groups (gp), each contained eight rats, according to the application of laser therapy and force magnitudes, as follows: Control low-force gp (CL); with (10–15 g) orthodontic force (*n* = 8). Control high-force gp (CH); using (50 g) orthodontic force (*n* = 8). Laser low-force gp (LL); using (10–15 g) orthodontic force plus PBM (*n* = 8). Laser high-force gp (LH); with (50 g) applied force plus PBM (*n* = 8).

**Fig. 1:**
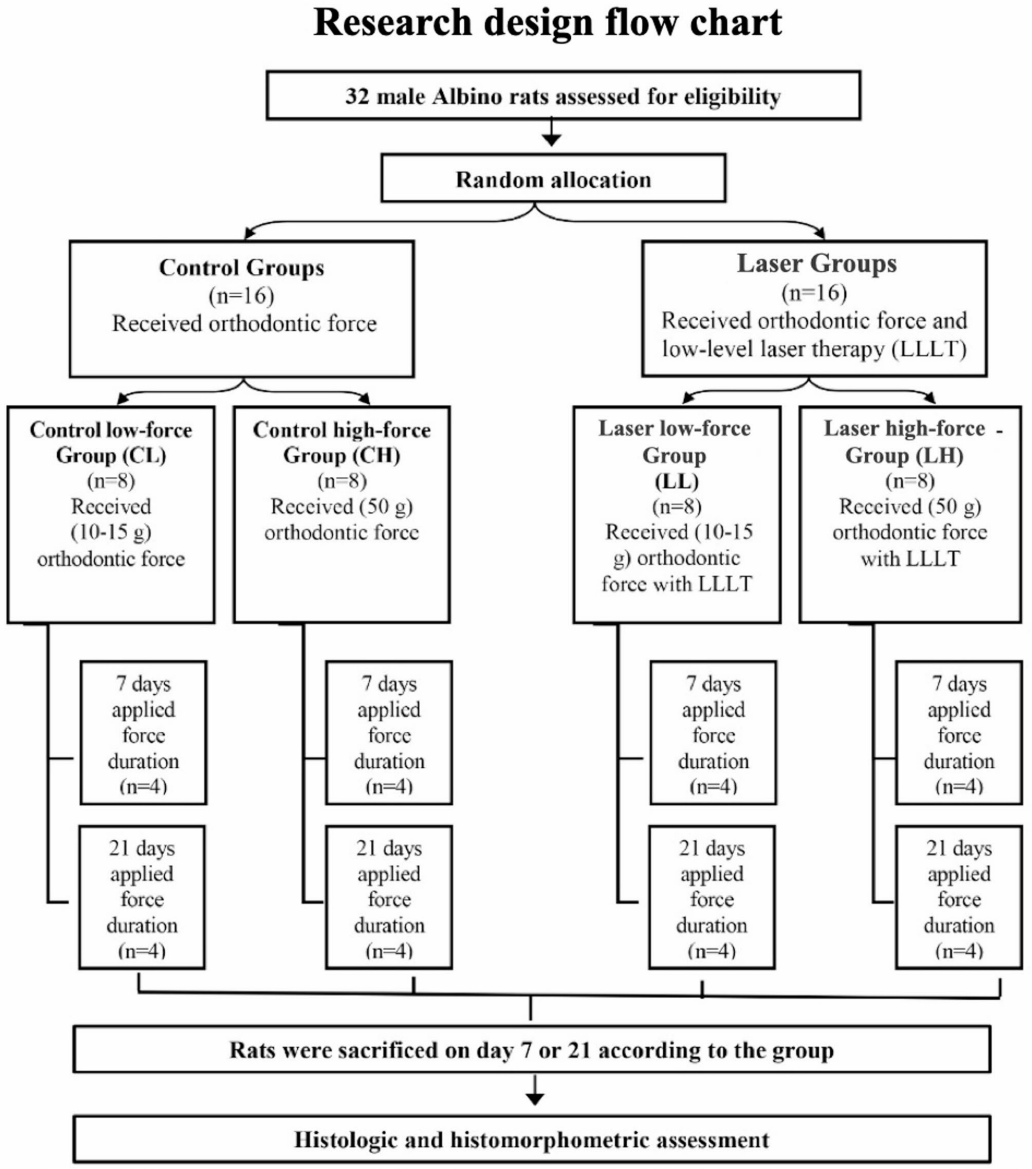
Flow chart showing research design

Each gp subsequently was divided into 2 subgroups each of 4 rats (*n* = 4) according to the duration of force application: 7-days and 21-days (Fig. 1).

#### Randomization technique

Simple randomization was conducted with equal allocation ratios [22]. The random allocation made by using a computer-generated random sequence of numbers to one of the four arms (control low-force, control high-force, laser low-force and laser high-force). The random sequence was made using computer-assisted random number generator software[23], and numbers of 8 rats were randomly chosen for each gp.

#### Blinding (masking)

Blinding (masking) was considered through all the steps of the present study [24]. The participant and all personnel who perform the study were unaware of the treatment assignment. The operator was masked during the animals’ allocation, but further masking of the operator was not possible till the sacrifice of the animal due to nature of the intervention. While histologic and histomorphometric assessment was done by an investigator blinded to the identity of the experimental subgroups. Moreover, data analysis was conducted in a blinded fashion.

### Experimental procedures

#### Anesthesia

Prior to the installation of orthodontic appliances, animals received general anesthesia by intramuscular injections of xylazine hydrochloride (30 mg/kg) and ketamine hydrochloride (70 mg/kg).

#### Orthodontic force application

An experimental model of OTM was fabricated in all the experimental animals using the methodology outlined in other papers [25, 26]. 

**Fig. 2:**
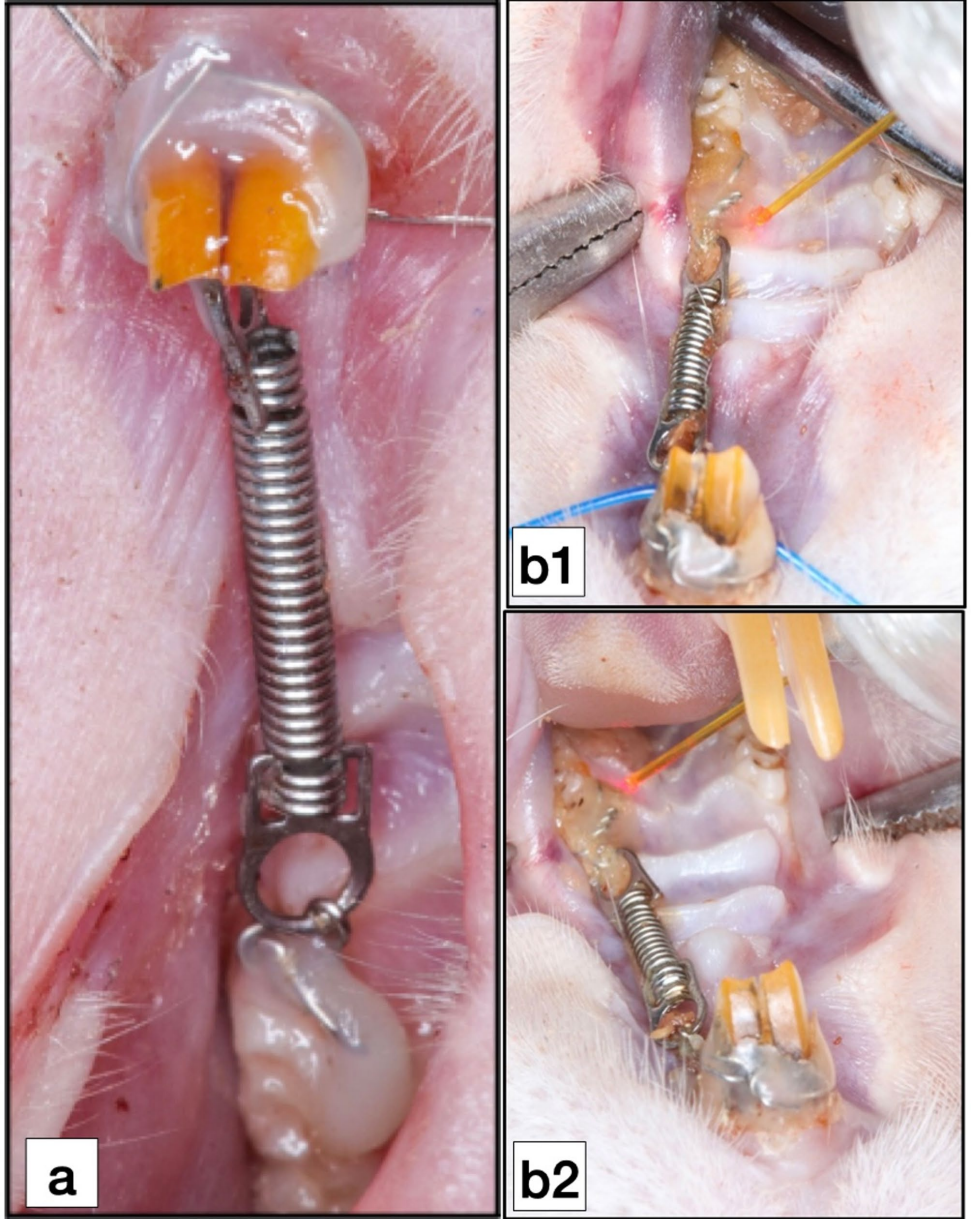
**a** Occlusal view of experimental orthodontic appliance in situ; **b** Laser irradiation using 980 nm diode laser; **b1**, Mesial-palatal point; **b2**, Distal-palatal point

The orthodontic appliance consisted of NiTi closed coil spring (10 mm length; 1.5 mm diameter) (DB Orthodontic Company, West Yorkshire, United Kingdom) fixed and stretched between the maxillary left first molar (M1) and maxillary incisors to exert orthodontic force of approximately 10–15 g and 50 g for the low-force gps and high-force gps, respectively. The force magnitude was adjusted according to the groups by the help of a force gauge (YDM corporation, HIGASHIMATSUYAMA, JAPAN). The appliance was left for 1 or 3 weeks, according to the euthanasia date, to produce appreciable tooth movement and root resorption (Fig. 2a).

#### Laser irradiation

The laser irradiation in LL and LH gps was conducted by a GaAlAs diode laser (Simpler Doctor Smile Dental Diode Laser, LAMBDA SPA, ITALY) (wavelength: 980 nm, output power: 100 mW). Oral mucosa around the M1 was irradiated every other day at four points (two points palatal and two buccal) with a continuous non-contact irradiating mode for 12 s for each point (energy of 1.2 J/point, total radiant energy of 4.8 j for net of the four points, fluence (energy density) of 16.9 J/cm^2^). The tip was kept 1 mm from the irradiated tissue. Total radiant energy were 4.8 j for the net of the four points. Beam spot size at target equal 0.071 cm^2^ with a total area of irradiation equal 0.284 cm^2^ [27]. (Fig. 2b).

#### Euthanasia

At the finished point of the experiment the experimental animals were anesthetized by intravenous injection with a lethal dose (100 mg/kg) of pentobarbital sodium [28]. 

### Tissue preparation for light microscopic examination and histomorphometry

The maxillae were dissected out and the surrounding epithelial and muscular components were eliminated. The obtained specimens were immersed in 10% neutral buffered formaldehyde and demineralized using trichloro-acetic acid, thereafter dehydrated in ascending concentrations of alcohol, cleared using xylene, infiltrated, and embedded in paraffin wax. 4-µm serial transverse sections at the cervical two thirds of maxillary left first molar’s roots were obtained and stained with H&E stain [29, 30]. The general histologic evaluation was conducted on all the roots except the mesial one (the largest root). The number and type of the evaluated roots are the same in all the gps.

Active root-resorptive foci were defined as resorptive cavities in which odontoclasts could be visible. Cells were defined as odontoclasts if they were multinucleated eosinophilic cells with round nuclei, either on the root surface or inside root resorption crater. On the other hand, formation of a basophilic layer of reparative cementum or cementoid tissue in the root resorption lacunae was determined as an indicator of the reparative events of the resorption foci.

### Histomorphometric analysis

Histomorphometric analysis was done using a computer program ImageJ 1.50e (ImageJ, National Institute of Health, USA). For each specimen, two sections, with the greatest expressive tissue events, from the cervical two thirds of the disto-buccal (DB) root, were chosen for histomorphometric assessment, from which root resorption percentage was calculated. These two sections were selected in standardized way after obtaining full root length sections, ordering them, chosing the cervical two thirds sections and chosing the two sections with the deepest total root resorption areas for statistical analysis.

#### Measurement of root resorption lacunae

The software ImageJ was utilized to calculate the percentage of radicular resorption craters for the histomorphometry [31, 32]. The examined region belongs to the DB root. To calculate the percentage of root resorption relative to the total area of hard root structures, a modified method of Vilela et al.[17] was used. The entire radicular area was first measured, putting into consideration an external virtual line that confined the resorption lacunae and continued the root outline, subsequently, the area of the pulpal area had been subtracted from the total radiular area to ascertain the area of the hard root structure excluding the pulpal cavity. The resorption craters were then measured, putting into consideration the same external virtual line which outlined the resorbed foci and continued the radicular outline, and the root resorption ratio to the total radicular area and the total percentage of root resorption were then calculated.

#### Capillaries count

**Fig. 3:**
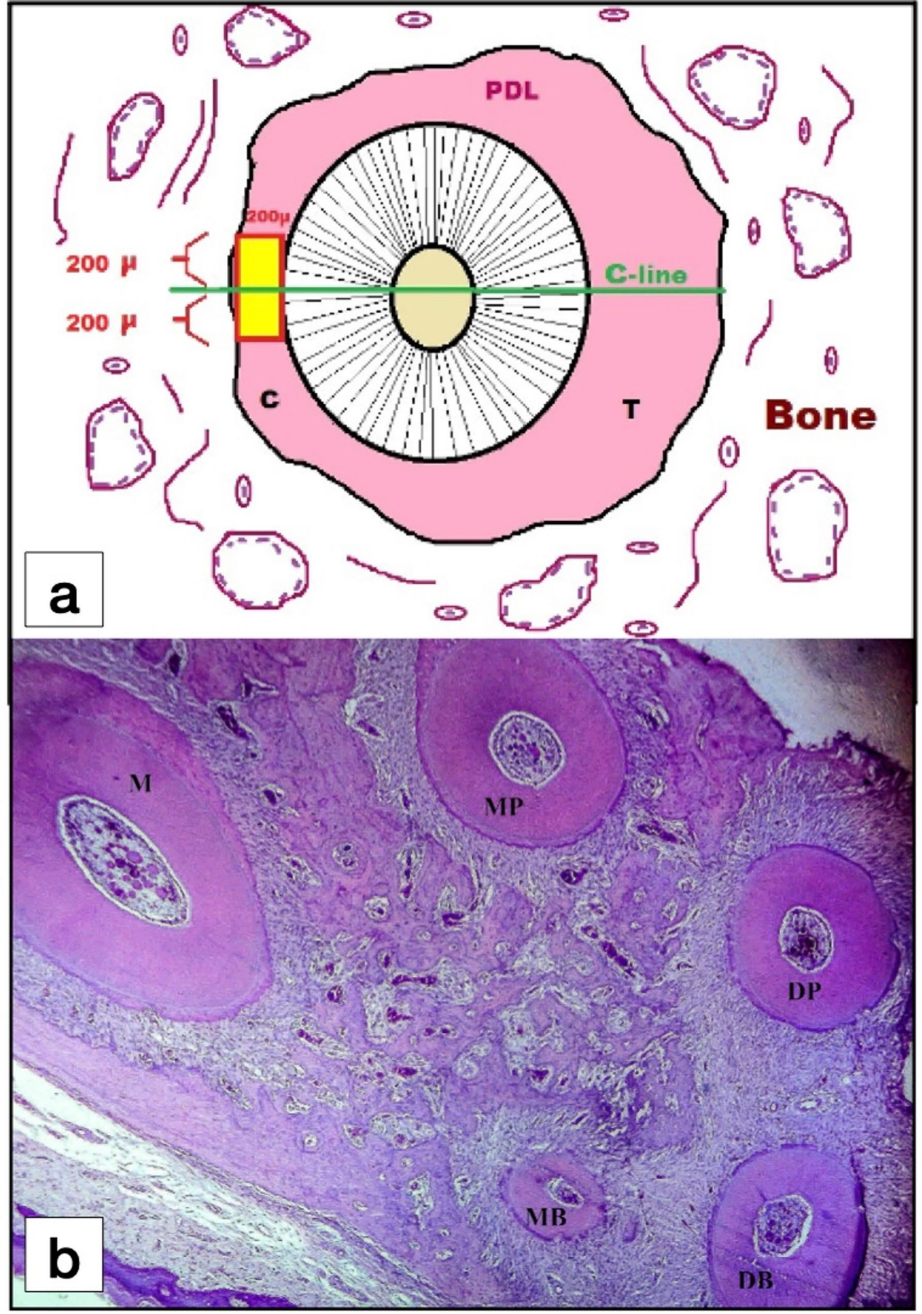
**a** Overlay was layered over the image of the cross section of the distal-buccal root to designate the area used for measurement. The line C is the mesial-distal line passing through the center of the distal-buccal root and parallel to the direction of tooth movement. A rectangular area of (400 × 200 µm^2^) was designated under 100-times magnification bisecting line C representing the region of interest for quantification; **b** Light micrograph (L.M) of cross section of the rat maxillary first molar at the cervical two thirds of the roots showing the distribution of the five roots; (H&E staining, x40). M, Mesial root; MB, Mesial-buccal root; DB, Distal-buccal root; MP, Mesial-palatal root; DP, Distal-palatal root; PDL, Periodontal ligament; C, Compression side of the PDL; T, Tension side of the PDL

The images at 100 times magnification power were imported to Adobe Photoshop CS6 Image Processing Software (Version 13, Adobe Systems Incorporated, San Jose, CA, USA). An overlay was layered over the image to outline the area needed for measurement [33]. First, the overlay was scaled to the size of the image using the 10 μm bar. Second, a mesio-distal line going through the center of the DB root and parallel to tooth movement direction was drawn (which is “line C”). A 400 μm tangent line was drawn touching the mesial root surface and bisecting and perpendicular to the line C (200 μm above and 200 μm below the line C). This line was used to draw a rectangle of 400 μm height and 200 μm width in the mesial PDL that touches the mesial root surface. The capillaries were counted within the designated rectangular area (400 × 200 µm^2^) using ImageJ software (Fig. 3).

### Statistical analysis

IBM SPSS software package version 22.0 (IBM Corp, Armonk, NY) was utilized for data analysis [34]. Outcome variables were tested for normality by Kolmogorov-Smirnov and Shapiro-Wilk normality tests. They indicated no significance in the distribution of the variables; hence, the parametric statistics were used. (Tables of Kolmogorov-Smirnov and Shapiro-Wilk tests found in supplementary files). Data description was done using mean and standard deviation. An alpha level was set to 5% with a significance level of 95%, i.e., statistical significance was predefined at *P* ≤ 0.05, and a beta error accepted up to 20% with a power of study of 80%. The outcome variables analyzed were the percentage of root resorption lacunae and the capillaries count.

For the comparison of outcome variables, F-test (ANOVA) was used For normally distributed quantitative variables, to compare between more than two gps. Post Hoc test (Tukey) was used For pairwise comparisons between the gps. Tables and bar charts were used accordingly.

## Results

### Tolerance of experimental procedures

Two experimental rats failed to survive. In the control-low force gp (CL), one rat died immediately after recovery from anesthesia after the fixation of the orthodontic appliance. In the laser low-force gp (LL), one rat died during recovery from inhalational anesthesia after one of the laser irradiation sessions. The two ratss were excluded from the study. The rest of the animals survived till the termination of the experiment in all the gps.

Except for the two excluded rats, the rest of the rats did not show any signs of general sickness all over the experimental duration. No deep mucosal infections, dehiscence, nor other detrimental consequences were seen in any of the rats in the experiment. Post-anesthetic drowsiness and decreased appetite transiently manifested during the first two days after the placement of the orthodontic device. Afterwards, eating had essentially returned to a normal rate indicating that the orthodontic appliance was not an interference. The orthodontic appliances were routinely checked during the experimental period. All the appliances were stable at completion of the experiment, with remaining forces exerted on the M1s.

### Reliability

One examiner performed all the measurements, intra examiner reliability was assessed by measuring the percentage of root resorption and number of blood vessels (BVS) at day 21 with about 6 to 8 h intervals between the measurements. The agreement between the quantitative data sets from the 1 st and 2nd measurements was assessed using Inter-Class Correlation Coefficient [35] (ICC) for number of the blood vessels, which was 0.952 with a 95% CI (0.872–0.983), which indicated an excellent agreement. Whereas, the ICC for root resorption percentage was 0.858 with a 95% CI (0.640–0.948), which indicated a good agreement (Table 1). Furthermore, the Bland-Altman test was used to assess the agreement between the two repeated measurements (test-retest reliability) for both root resorption measurements and blood count. The mean difference (bias) between the two measurements for root resorption was − 0.01 (*p* = 0.795), indicating excellent agreement, while for blood count it was 0.10 (*p* = 0.678), indicating good average agreement (The table and figures of the Bland-Altman test are found in supplementary files).

**Table 1:** Intra-rater reliability of measured variables

Variables	Intra-rater reliability	
	Interclass Correlation Coefficient (ICC)	95% CI
Number of BVS^ͳ^	0.952	0.872–0.983
Percentage of root resorption^ͳ^	0.858	0.640–0.948

^ͳ^ Based on a two-way mixed model using an absolute agreement

### The results derived from this study include the following

#### Histological results

**Fig. 4:**
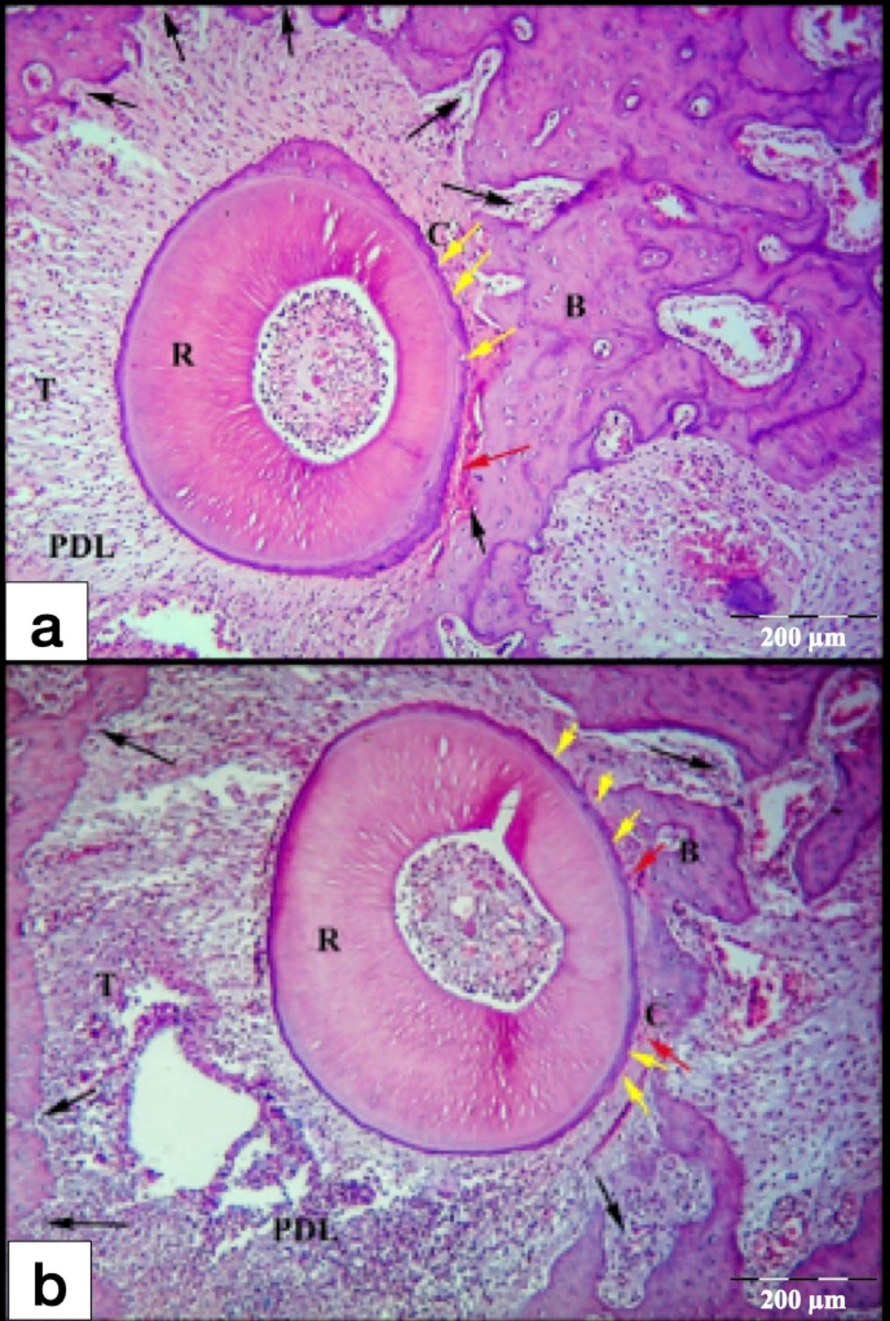
L.M of cross sections of the distal-buccal (DB) root in the rat maxillary first molar (M1) at day 7. **a** Control low-force group (CL); **b** Control high-force group (CH), showing typical compression of the PDL at the compression site (C) on the mesial side of the root, and stretching of the PDL at the tension site (T) on distal side. Note: An area of initial hyalinization at compression side (Red arrow), rough surface of the root (yellow arrows) and resorption foci in the alveolar bone (black arrows). R, Root; PDL, Periodontal ligament; B, Bone. (H&E staining, x100)

**Fig. 5:**
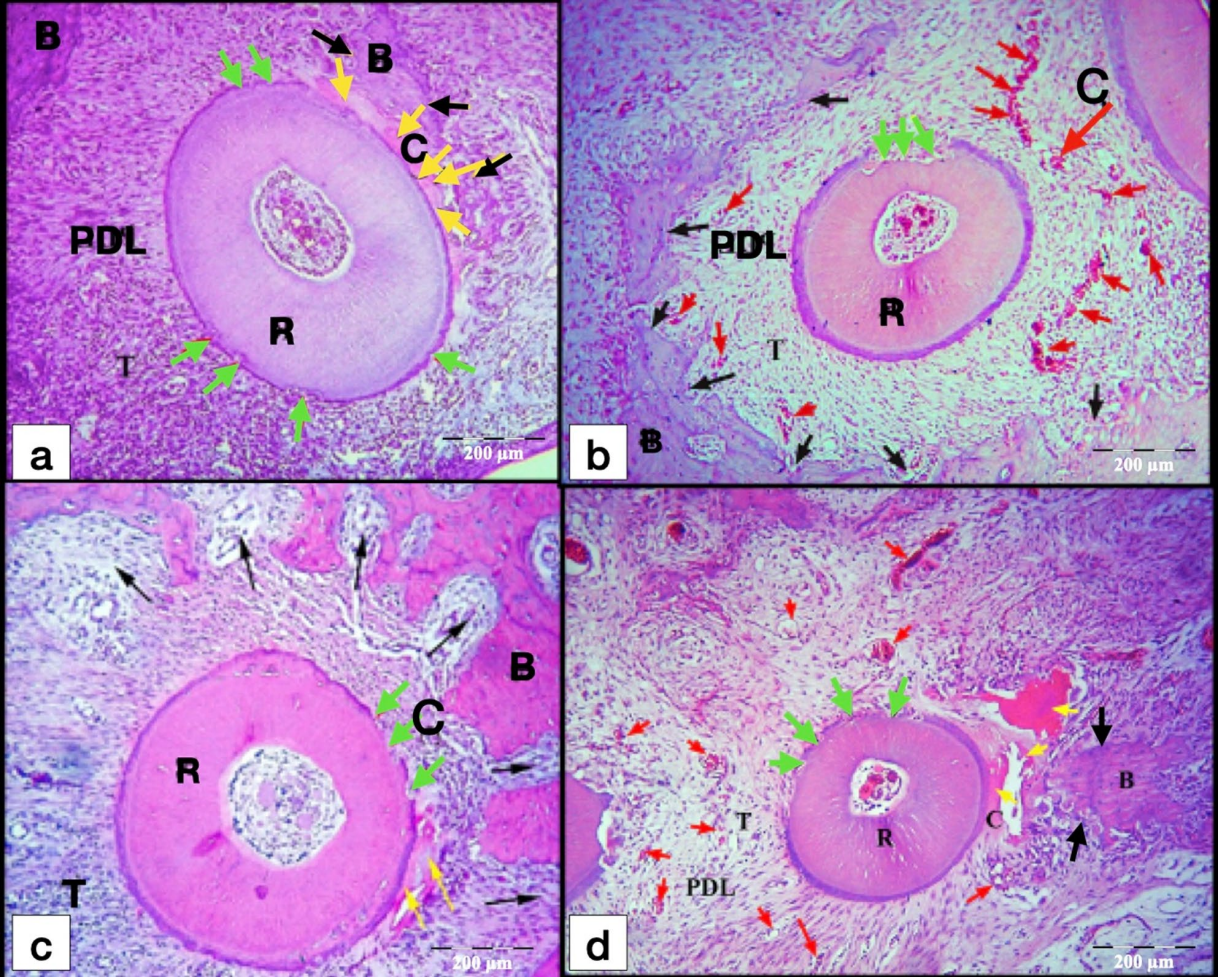
L.M of cross sections of roots in the M1 at day 7. **a** DB root in laser low-force group (LL); **b** mesial-buccal (MB) root in LL group; **c** DB root in laser high-force group (LH); **d** MB root in LH group, showing hyalinization of the PDL (yellow arrows) at the compression site (C) with extensive resorption of the alveolar bone (black arrows) near the hyalinized tissues. Widespread extensive cellular infiltration is noted in the PDL. Obvious widespread blood vessels engorged with RBCs (red arrows) are noted in the PDL especially at the C site. Note: Rough surface of the root with minor root resorption foci (green arrows). B, Bone; R, Root; PDL, Periodontal ligament; T, Tension site of PDL. (H&E staining, x100)

**Fig. 6:**
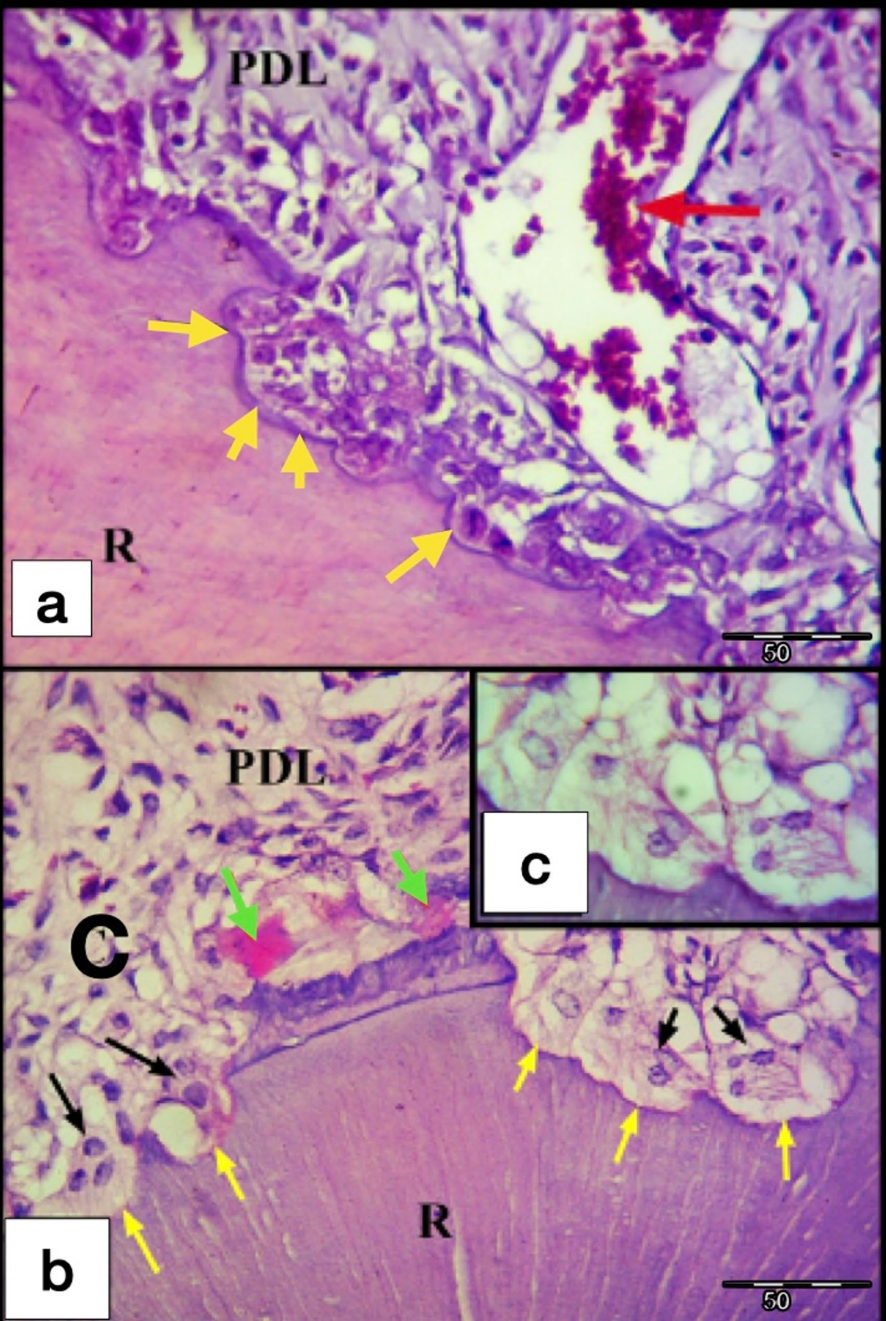
L.M of cross sections of the DB root of M1 at day 7. **a** LL group showing root resorption areas some of them are covered by a thin layer of newly formed cementum deposition (yellow arrows). Note the quite large blood vessel engorged with RBCs (red arrow) found in the PDL near the resorption areas. **b** LH group showing areas of root resorption (yellow arrows) with the presence of active odontoclasts (black arrows) resorbing the dentine. Note: The presence of remnants of hyalinized tissues (green arrows) in the PDL adjacent to the root resorption areas. **c** odontoclast in situ. R, Root; PDL, Periodontal ligament; C, Compression side of the PDL (H&E staining, **a**&**b**: x400, **c**: x1000)

After 7-days of exerted orthodontic force, the control gps (CL and CH) showed initial hyalinization of the PDL at the compression side, in addition to minor areas of bone and root resorption in the tooth movement direction (Fig. 4). On the other hand, laser gps (LL and LH) showed mature areas of hyalinization of the PDL which were detected in few sections, some of them appeared as remnants of necrotic tissues after being invaded by the odontoclasts. Besides, active areas of bone and root resorption were easily detected in the laser gps, mainly in the direction of the tooth movement (Fig. 5). Also, odontoclasts were frequently observed in the root resorption craters in the laser 7-days subgroups, indicating active root resorption processes (Fig. 6). Almost all the sections in the laser 7-days subgroups showed wide spread blood vessels engorged with RBCs in the PDL space (Fig. 5). For the LL 7-days subgroup, some of the root resorption craters were covered by thin layer of reparative cementum depositions, which were less frequently found in the LH subgroup, indicating for early start of the reparative events (Fig. 6a).

**Fig. 7:**
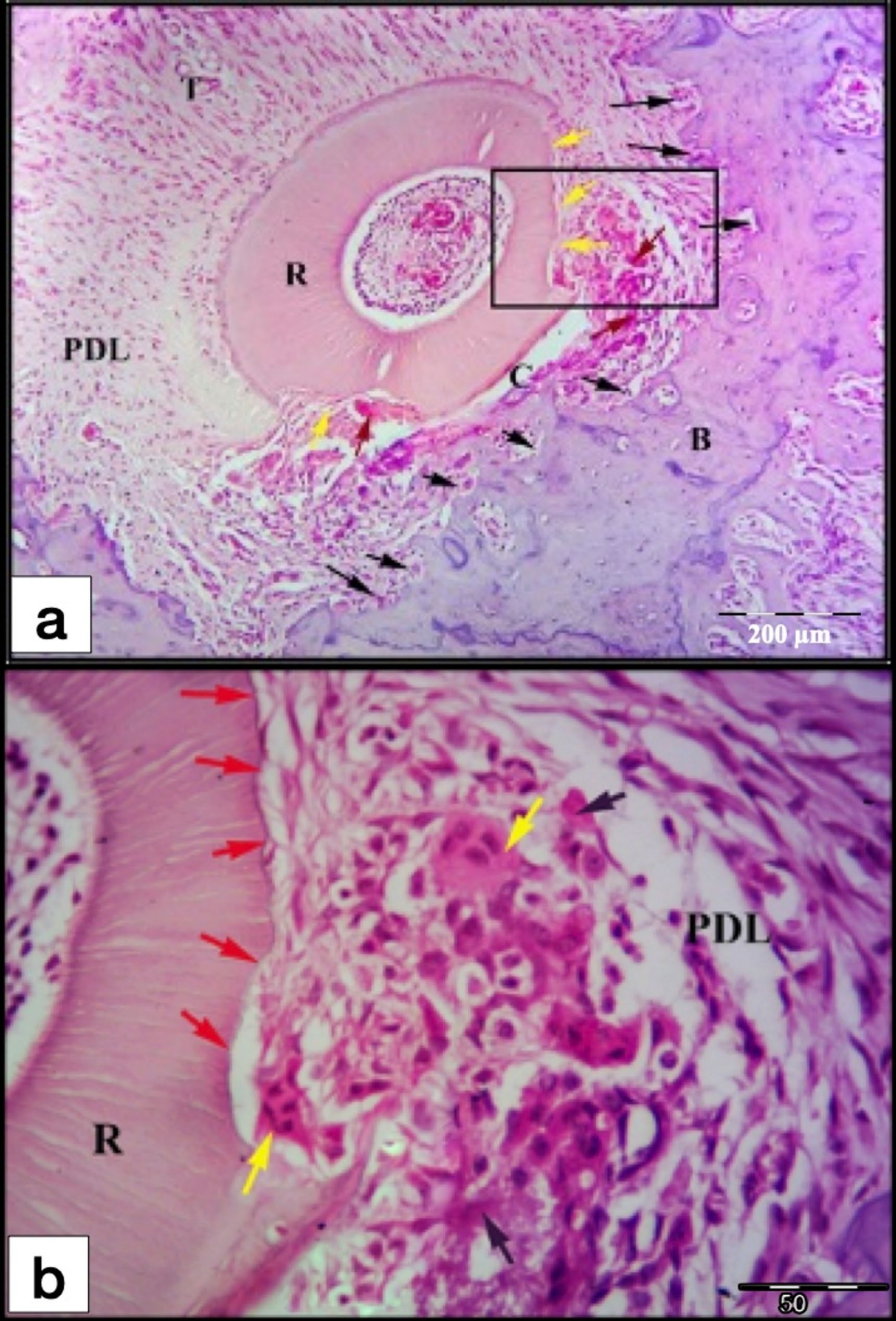
L.M of a cross section of the DB root of M1 (CL group at day 21). **a** Remnants of hyalinized tissues (red arrows) and irregular distribution of the PDL fibers at the compression site (C) of the PDL, also stretching of the PDL at the tension site (T) on distal side. Note: Areas of extensive root resorption (yellow arrows), and widespread resorption foci in the alveolar bone (black arrows). b Higher magnification showing extensive root resorption area (red arrows) with odontoclasts (yellow arrows) resorbing the root structure and removing the remnants of the hyalinized tissues (black arrows). R, Root; PDL, Periodontal ligament; B, Bone. (H&E staining, **a**: x100, **b**: x400)

**Fig. 8:**
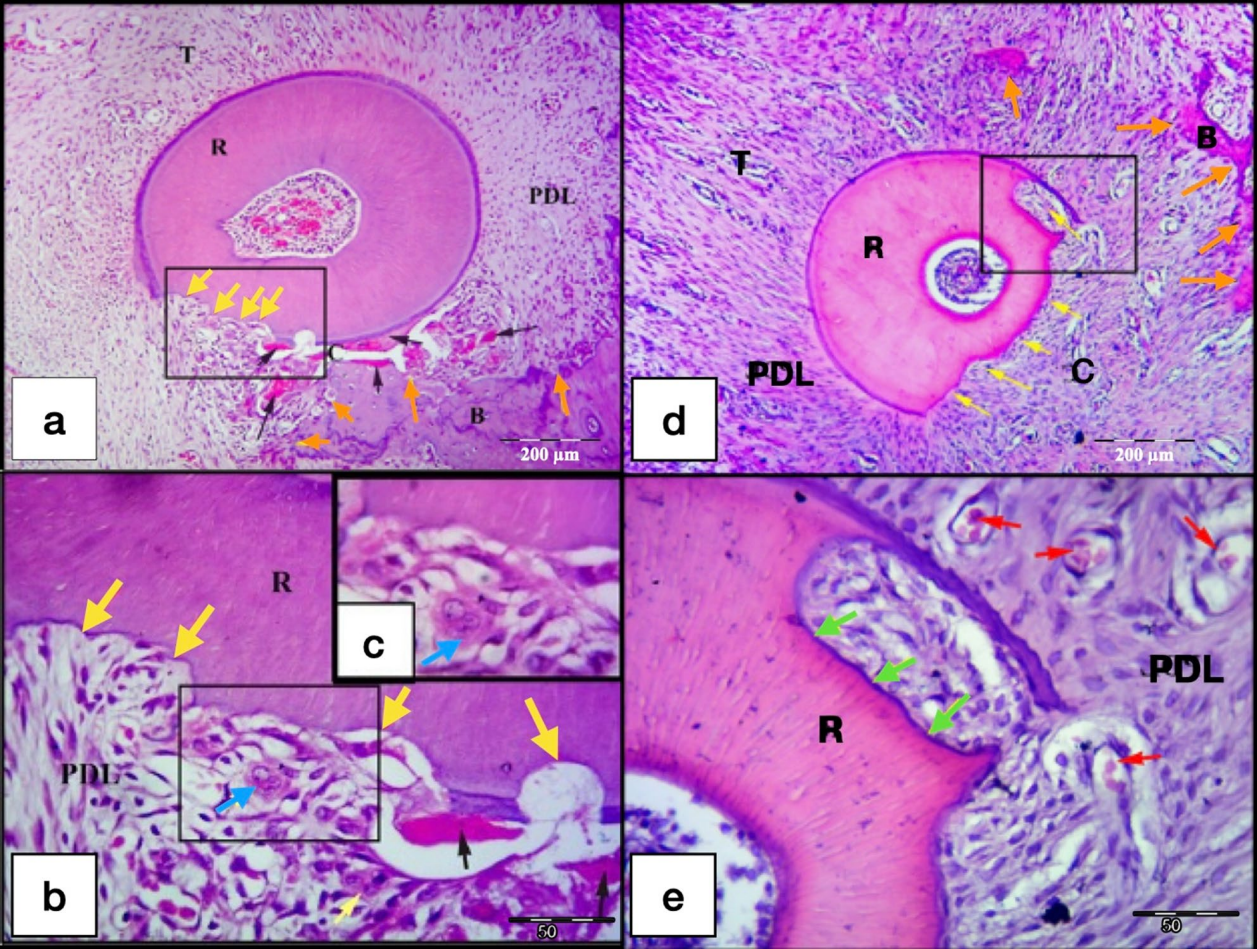
L.M of cross sections in the DB root of M1 (CH group at day 21). **a** showing remnants of hyalinized tissues (black arrows) at the compression site (C), extensive root resorption (yellow arrows) and start of resorption of the alveolar bone (orange arrows) near the hyalinized tissues of the PDL. **b**&**c** Higher magnifications of the inset of Figure (**7a**) showing extensive root resorption area (yellow arrows) with odontoclasts (blue arrows) resorbing the root structure and removing the remnants of the hyalinized tissues (black arrows). **d** showing large area of extensive root resorption (yellow arrows) and irregular distribution of the PDL fibers at the compression site (C) of the PDL, but they are more organized than control 7-days subgroups. **e** Higher magnification of the inset of the previous Figure(**7d**) showing extensive root resorption area with very minimal reparative cementum deposition (green arrows). Note: The presence of small blood capillaries near the resorbed area (red arrows). R, Root; PDL, Periodontal ligament; B, Bone; T, Tension site of PDL. (H&E staining, **a**&**d**: x100, **b**&**e**: x400, **c**: x1000)

After 21-days of exerted orthodontic force, the control gps (CL & CH) showed irregular orientation of the PDL fibers, but they were more organized than the control 7-days subgroups. Remnants of hyalinized tissues were frequently seen in the CH 21-days subgroup and were less frequently detected in the CL subgroup (Figs. 7 and 8a and b). Most of the specimens showed extensive areas of root resorption, most of them extended to the dentine layer (Figs. 7 and 8). No reparative cementum deposition was found in the control subgroups, except for few sections in the CH subgroup showed thin layer of reparative cementum (Fig. 8d&e). Odontoclasts were frequently observed in the CL and CH subgroups indicating still active root resorption processes (Figs. 7 and 8a-c). Few blood capillaries were seen in the compression site of the periodontal ligament. Moreover, extensive bone resorption was observed.

**Fig. 9:**
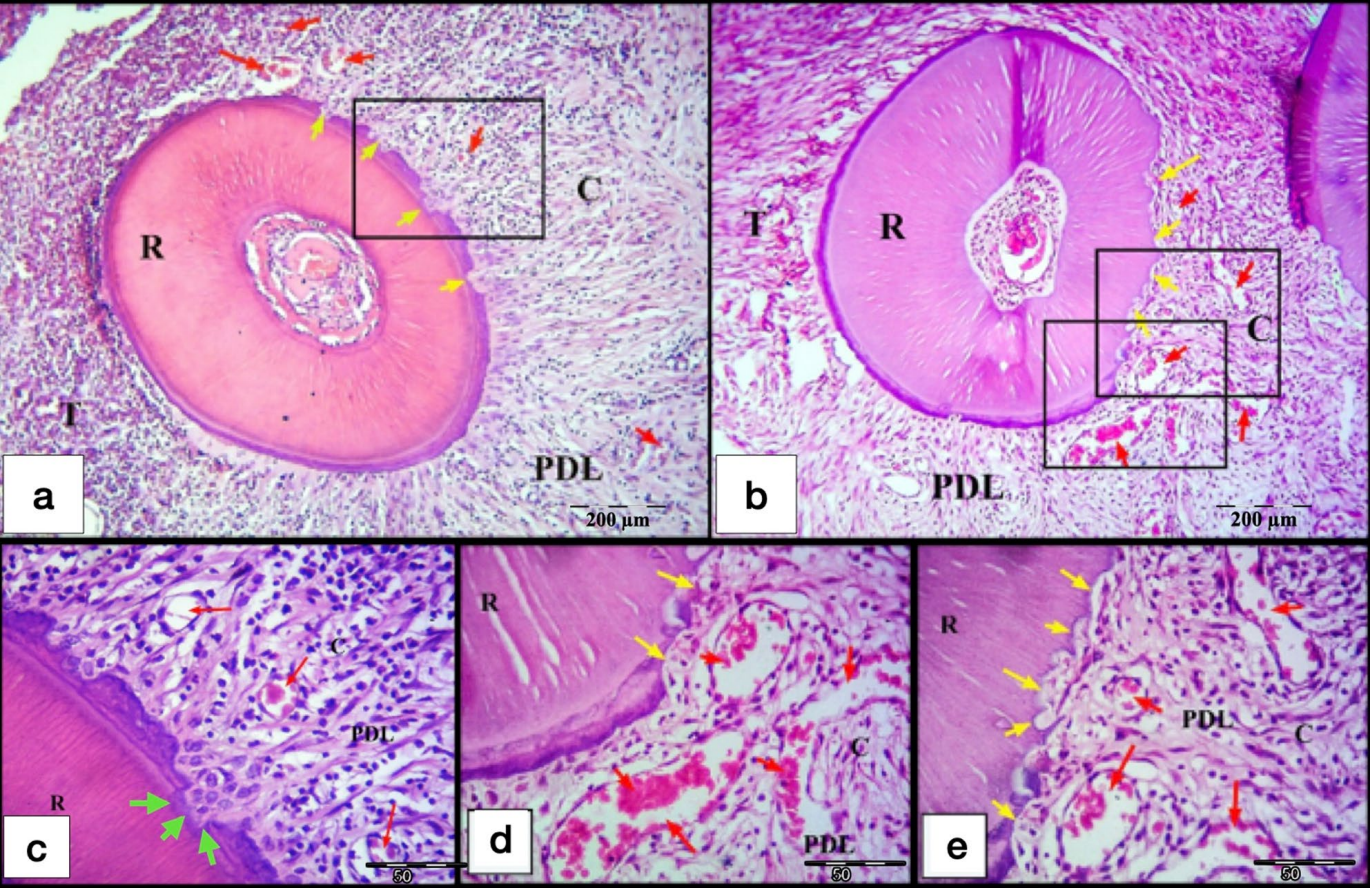
L.M of cross sections of the DB root in M1 (LL group at day 21). **a**&**b** show minor areas of root resorption (yellow arrows) mainly at the compression site (C) of the PDL, with absence of interradicular alveolar bone. Rearrangement of the PDL fibers at the compression site (C) and widespread blood vessels engorged with RBCs (red arrows) are noted in the PDL.**c** Higher magnification of the inset of the Fig. 8a showing minor root resorption foci covered by reparative cementum deposition (green arrows). Note: Widespread blood capillaries near the resorption foci (red arrows). **d**&**e** Higher magnification of the insets of the previous Fig. 8b showing increase in the blood vasculature crowded with RBCs (red arrows) of the PDL near areas of root resorption (yellow arrows). R, Root; PDL, Periodontal ligament; T, Tension site of PDL. (H&E staining,, **a**&**b**: x100, **c**,** d**&**e**: x400)

**Fig. 10:**
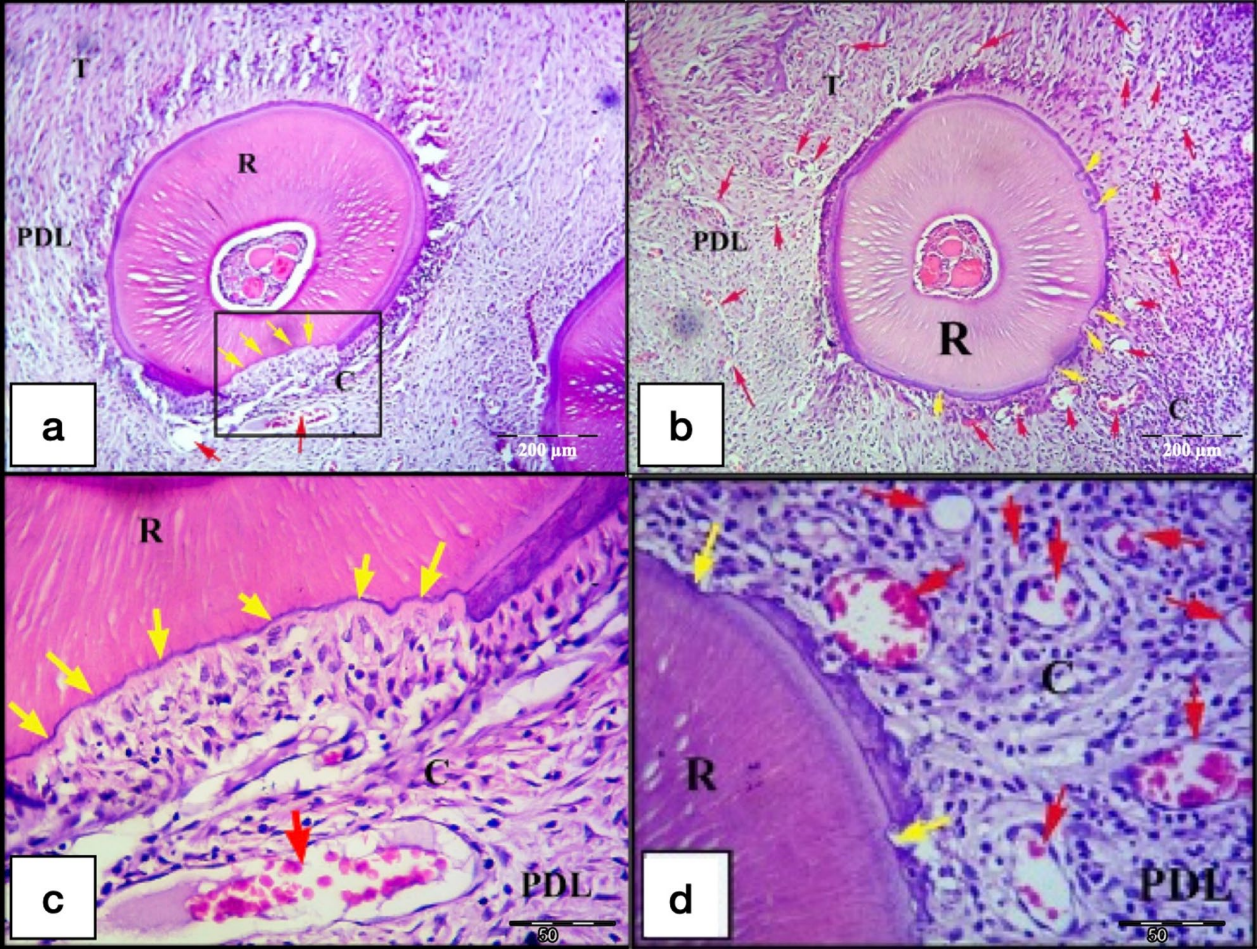
L.M of cross sections of the DB root in M1 (LH group at day 21). a&b show areas of root resorption (yellow arrows) at the compression site (C) of the PDL, with extensive resorption of almost whole the interradicular alveolar bone at this section level. Note: Irregular arrangement of the PDL fibers at the compression site (C) and the presence of a quite large blood vessel engorged with RBCs (red arrow) near the resorption crater. **c** Higher magnification of the inset of the previous Fig. 9a showing root resorption area with minimal reparative cementum deposition (yellow arrows). Note: The large blood vessel (red arrow) found in the PDL near the resorption crater. **d** shows increase in the blood vasculature crowded with RBCs (red arrows) of the PDL near areas of root resorption (yellow arrows) at the compression site (C) of the PDL, also minimal deposition of reparative cementum is seen in some of the resorption craters. R, Root; PDL, Periodontal ligament; T, Tension site of PDL. (H&E staining, **a**&**b**: x100, **c**,** d**: x400)

On the other hand, laser gps (LL and LH), at day 21, showed proper re-arrangement of the PDL fibers especially for the LH gp. Root resorption areas, confined to the cementum only or extended to the dentin, were detected, especially in the LH 21-days subgroup. Most of the root resorption craters were covered by reparative cementum, especially for the LH subgroup which showed the most distinct cementum depositions over all the other control and laser subgroups. No odontoclasts nor hyalinized tissues were detected in all the lased specimens. All the sections in the laser groups showed wide spread of blood vessels engorged with RBCs in the PDL space (Figs. 9 and 10). The most extensive bone resorption was detected in the laser 21-days subgroups.

#### Histomorphometric results

##### The percentage of OIIRR

**Fig. 11:**
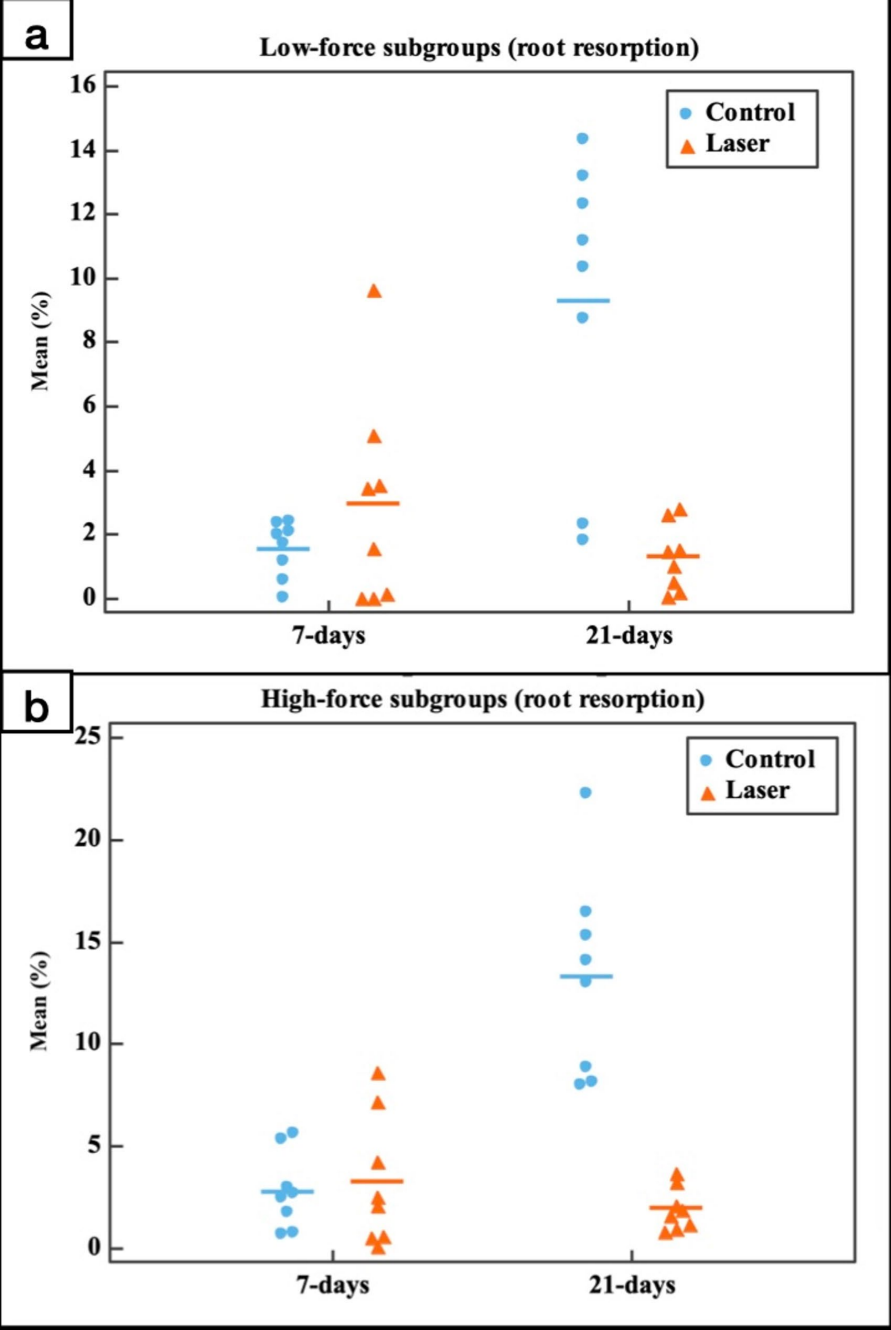
Dot plot representing the percentage of root resorption comparing the control group with the laser group in the different experimental periods. **a** Low-force groups, **b** High-force groups

The analysis showed no significant difference in the degree of OIIRR between the control gps (CL (1.56 ± 0.87%) and CH (2.81 ± 1.86%)) and the laser gps (LL (2.97 ± 3.31%) and LH (3.28 ± 3.20%)) at day 7 (*p* = 0.982 & *p* = 1.000 for low-force and high force subgroups, respectively). However, There was significant reduction in the amount of OIIRR in the laser gps (LL (1.31 ± 1.04%) and LH (1.98 ± 1.05%)) over the control gps (CL (9.29 ± 4.77%) and CH (13.29 ± 4.93%) and at day 21 (*p* < 0.001 for both low-force and high force subgroups). Moreover, the amount of OIIRR significantly increased (*p* < 0.001) from day 7 to day 21 in the control gps (CL and CH), while the laser gps (LL and LH) revealed no statistically significant change in the degree of OIIRR from day 7 to day 21 (*p* = 0.958 and *p* = 0.989 for the LL subgroups and LH subgroups, respectively). Besides, the high-force subgroups showed an increase in the amount of OIIRR over the low-force subgroups for both the control and laser gps. However, this increase in the amount of OIIRR was statistically not significant (*p* = 0.991 for the control 7 days subgroups, *p* = 0.171 for the control 21 days subgroups, *p* = 1.000 for the laser 7 days subgroups and *p* = 1.000 for the laser 21 days subgroups) (Fig. 11; Table 2).

**Table 2:** Comparison between the different studied groups according to percentage of root resorption

	Control				Laser			
	Low		High		Low		High	
	7 (*n* = 8)	21 (*n* = 8)	7 (*n* = 8)	21 (*n* = 8)	7 (*n* = 8)	21 (*n* = 8)	7 (*n* = 8)	21 (*n* = 8)
Mean	1.56^c^	9.29^ab^	2.81^bc^	13.29^a^	2.97^c^	1.31^c^	3.28^c^	1.98^c^
±SD.	0.87	4.77	1.86	4.93	3.31	1.04	3.20	1.05
F(*p*)	16.171^*^(< 0.001^*^)							

F: F for ANOVA test, Pairwise comparison bet. each 2 subgroups was done using Post Hoc Test (Tukey)

*: Statistically significant at *p* ≤ 0.05

Means with Common letters are not significant (i.e. Means with Different letters are significant). *N* = 8 in each subgroup

##### Capillaries count

**Fig. 12:**
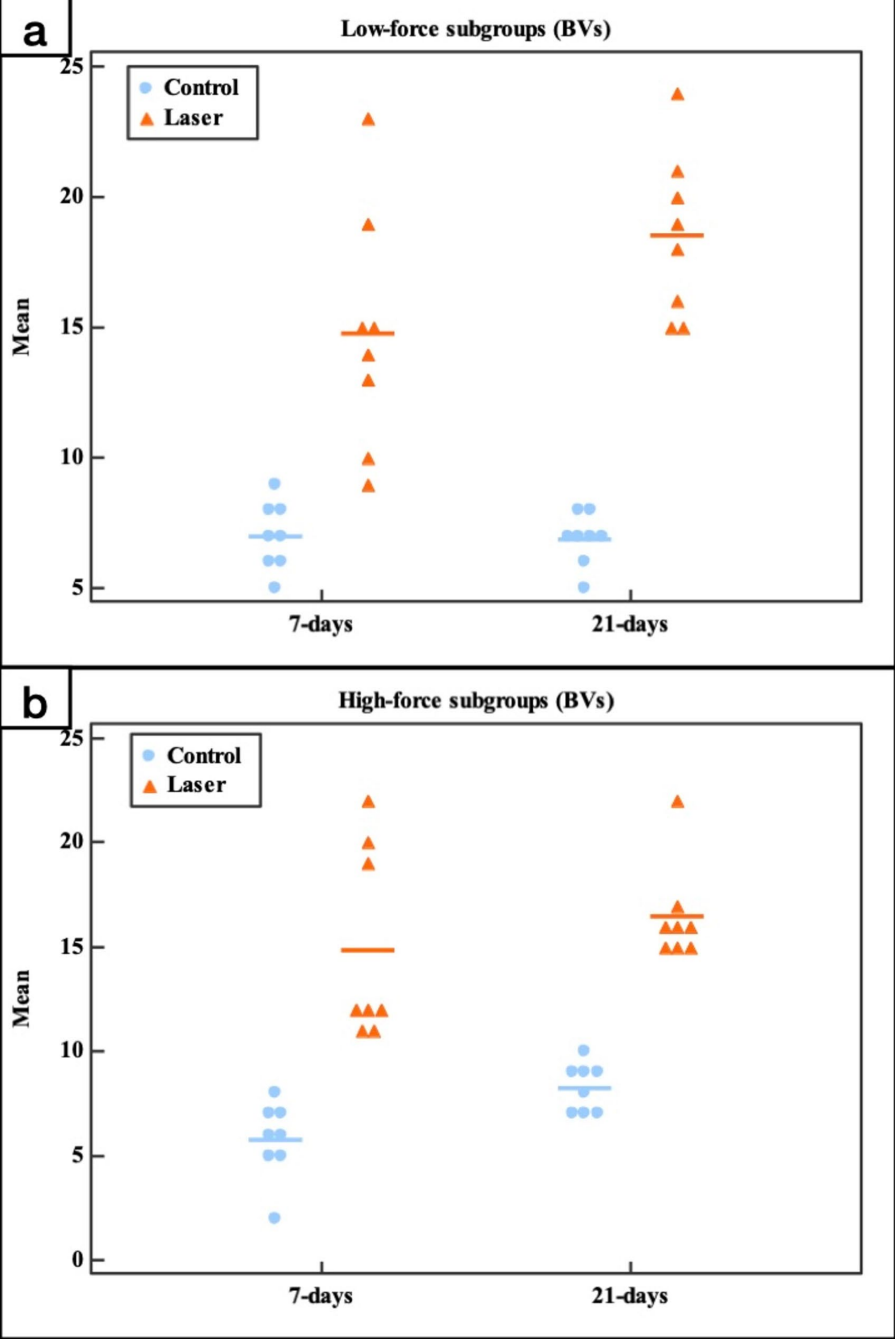
Dot plot representing the number of the blood vessels (BVs) comparing the control group with the laser group in the different experimental periods. **a** Low-force groups, **b** High-force groups.

PBM significantly increased blood capillaries count in the laser gps (LL (14.75) and LH (14.88)), in comparison to the control gps (CL (7.0 ± 1.31) and CH (5.75 ± 1.83)) at day 7 (*p* < 0.001). Also, PBM significantly increased the number of blood capillaries in the laser gps (LL (18.50 ± 3.16) and LH (16.50 ± 2.33)), in comparison to the control gps (CL (6.88 ± 0.99) and CH (8.25 ± 1.16)) at day 21 (*p* < 0.001). Besides, there was no statistically significant change in the blood capillaries count from day 7 to day 21 in all control gps (CL (*p* = 1.000) & CH (*p* = 0.652)). In the laser gp, it was found that the longer the experimental period the higher the number of blood capillaries for both LL and LH gps. However, this increase was statistically not significant (*p* = 0.166 and *p* = 0.945 for the LL and LH subgroups, respectively). Besides, there was no statistically significant difference in the capillaries count between the low-force and high-force subgroups in both control gps (*p* = 0.987 at 7days & *p* = 0.977 at 21 days) and laser gps (*p* = 1.000 at 7days & *p* = 0.851 at 21 days) (Fig. 12; Table 3).

**Table 3:** Comparison between the different studied groups according to capillaries count

	Control				Laser			
	Low		High		Low		High	
	7 (*n* = 8)	21 (*n* = 8)	7 (*n* = 8)	21 (*n* = 8)	7 (*n* = 8)	21 (*n* = 8)	7 (*n* = 8)	21 (*n* = 8)
Mean	7.0^b^	6.88^b^	5.75^b^	8.25^b^	14.75^a^	18.50^a^	14.88^a^	16.50^a^
±SD.	1.31	0.99	1.83	1.16	4.56	3.16	4.61	2.33
F(*p*)	25.528^*^(< 0.001^*^)							

F: F for ANOVA test, Pairwise comparison bet. each 2 subgroups was done using Post Hoc Test (Tukey).

*: Statistically significant at *p* ≤ 0.05.

Means with Common letters are not significant (i.e. Means with Different letters are significant). *N* = 8 in each subgroup.

## Discussion

This experiment conducted induced OTM in an animal model to clarify the potential impacts of PBM as a noninvasive technique for suppressing or decreasing OIIRR. The study intervals of 7- and 21-days were defined based on literature reports [16, 36]. The average life expectancy of rats is approximately 2 years, whereas the worldwide average life expectancy in human population is approximately 72.5 years [37]. Converting rat age to human age, a 21-days-long period for rats is approximately equivalent to two years for humans, which is the typical length of time required for most of orthodontic treatment [38]. Besides, according to a prior research by Hellsing and Hammarstrom[39], definitive resorption lacunae form within one week of the orthodontic force exertion. Similarly, Jäger et al.[40] demonstrated that the healing of root resorption sites began 9 days following appliance placement. Besides, both clinical and animal reports revealed the presence of many phases in orthodontic tooth movement [41]. Van Leeuwen et al.[42] found that reaching the so-called ‘linear phase’, where actual tooth movement happens, requires from few days to few weeks in rodents. Consequently, investigations aiming to describe the biological events in the linear phase of tooth movement must have a minimum experimental time of 2 weeks. So, an experimental duration of 21-days seems sufficient to iduce resorption process, reach the linear phase and begin of actual root reparative events for proper assessment.

On the other side, a study duration of 7-days looks much better to assess the effect of PBM on osteoclasts and their activities before the actual start of normal reparative processes for the root resorption. This observation was further corroborated by findings documented by Yokoya et al.[43] who revealed a considerable constant rise in the osteoclast count on the compression site of the periodontal ligament from day 1 to day 7 applying of orthodontic forces. Moreover, Choi et al.[44] demonstrated that alveolar bone remodelling in rats may begin as early as day 8. So, it was supposed that by day 7 we might be able to observe and investigate the root resorption events before the actual start of bone formation and root repair activities.

The animals were subjected to two force magnitudes: a relatively light-force of 10–15 g and a heavier force of 50 g. Past reports using a rodent model to assess the OIIRR utilized orthodontic force ranging from 10 to 100 g [45, 46]. In the current investigation, the choice of the light-force was based on prior reports done by Kohno et al.[47] and Gonzales et al.[46] who suggested that the optimal force magnitude for OTM of the maxillary molar in rats is around 10 g. Thus, the choice of the 10 to 15 g was made to approximate the optimum force magnitude used for OTM in human for better reasonable comparisons between rats and humans. For the heavier force magnitude, The magnitude of 50 g was selected according to data from other investigations [15, 48]. Given that the human molar is over 20 times bigger than a rat molar, the 50 g exerted by coil spring used for a rat should equate to 1 kg for a human molar, that is believed as a heavy force [49]. We also used the heavy force because we considered the relatively short experimental duration and aimed to cause significant resorption lacunae rather than smooth concavities, in order to represent the possible early and late differences between the 50 g groups, and to provide adequate comparison between the light- and heavy-force groups.

The optimal wavelength for biostimulation is between 400 and 1000 nanometers (in the red or near-infrared to visible light spectrum) [50–53]. Since infrared light is poorly absorbed by haemoglobin and water, beams at these wavelengths can spread more deeply into the tissues [52]. According to the research of Hamblin and Demidova[54], Wavelengths between 600 and 700 nm are effective for superficial tissue treatment, whereas wavelengths beyond 780 nm and below 1000 nm are appropriate for deeper tissue treatment because of enhanced optical penetration depths. Consequently, a 980 nm infrared light-emitting laser was used to achieve the objective of this research, which is to activate bone and periodontal ligament cells located underneath soft tissues and deeper inside the alveolar process [55]. 

It is extremely difficult to determine the optimal light dosage (in terms of energy density “fluence”) for a certain medical condition [54]. Several published research have revealed the beneficial effects of photo-biostimulation therapy with doses range between 2 and 54 J[56–58]. Thus, we chose a total energy dose of 4.8 j for PBM in the current study to stay within this range.

In the current study, we applied the laser irradiation 48-h intervals, and with this time, we could study the effect of the light during different phases of the tooth movement. This laser application protocol was based on previous literature report [20]. Previous study of Ekizer et al.[53] applied the laser irradiation once a day for ten sessions during only the first ten days of a twenty-one days experiment. In the present study, we preferred to deliver laser irradiation on every other day, for seven to eight sessions for the 21-days subgroups and 3–4 sessions for the 7-days subgroups, to minimize the risk of too much times of inhalational anesthesia (required for the laser irradiation sessions) in a short period, and to assess the effect of the laser over a longer period of the experiment.

In the current study a slight rise in the amount of root resorption was detected in the laser gps over the control gps at day 7, but this rise was statistically not significant (*p*> 0.05). It has been reported that the number of osteoclasts increases substantially from day 1 to day 7 following orthodontic force application, then drops significantly by day 14 [43]. According to Passarella and Karu [59], the mitochondrial cytochrome C oxidase absorbs red and near infra-red light and is responsible for effects of photo-biomodulation, resulted in a rise in ATP production and improvement of the potential cell activities. Due to the fact that osteoclasts are multinucleated cells with a high level of mitochondrial activities, they become instantly influenced by the photobiomodulation.

So, it seems that the first week of the force application includes a rise in the osteoclasts’ count and the start of root and bone resorption activities, while the reparative events of the root and the bone remodeling do not start yet at this time under normal circumstances. Because it is widely believed that PBM accelerates the existing biological events in the tissue [60], it is logical that the PBM may slightly increase, or at least may not decrease, the amount of root resorption during the first week, because the osteoclastic activities are predominant at this time.

Altan et al.[20] suggested that PBM advances or accelerates the entire tissue response. They found a rise in the counts of osteoblasts and fibroblasts, as well as osteoprotegerin immunoreactivity, that may be considered components of the formation process, at day 11 after laser irradiation. This may explain why the increase in amount of root resorption was statistically not significant at day 7 in our study and day 11 in their study, despite the increased number of osteoclasts and odontoclasts with PBM reported by them in the first 11 days. In addition, the early detection of the initial reparative events with PBM by Altan et al.[20] might explain the presence of initial reparative cementum deposition covering root resorption lacunae of some of our specimens of the laser gps (especially the low-force laser gp), while none of the control gps’ specimens showed any reparative cementum deposition at this time. Also, this might explain our observations that revealed the early detection of mature hyalinized tissues with start of the removal of these necrotic tissues in the laser gps at day 7, while the control gps showed only initial formation of the hyalinized tissues without an obvious removal process of these tissues at this time.

In contrast, the current investigation revealed that PBM significantly decreased the amount of OIIRR in the low- and high-force subgroups at day 21. The difference between the control gps and laser gps at day 21 was statistically highly significant (*p* < 0.001). This observation coincides with prior reports that indicated the positive impacts of PBM in reducing OIIRR [15, 18, 48]. 

Suzuki et al.[15] did the best job of explaining how the PBM could reduce OIIRR. They claimed that after laser irradiation, the elevation in osteoclastic activities and RANKL and TRAP expression could result in faster loss of bone area at the compression side, which would then cause the force at the root/PDL/bone interface to drop quickly. The quick drop in the force at the PDL means that less hyalinized necrotic tissues are generated. This lowers the OIIRR since the OIIRR mostly occurs as a result of the process of getting rid of the hyalinized tissues.

In the current study, it was found that the hyalinization of the periodontal tissues at the compression side was accelerated by the PBM. Thus, the hyalinized tissues were completely formed at day 7 and the process of removal of hyalinized tissues had also been started at this time in the laser gps, while the control gps showed only severe over-compression of the periodontal ligament at the compression side with pyknotic nuclei (as signs of initial hyalinization process) at day 7. On the other hand, hyalinized tissues were completely removed in all the laser gps’ specimens at day 21, with better reorganization of the periodontal ligament fibers at the compression side and marked removal of almost whole of the inter-radicular bone facing the compression site of the PDL. In contrast, many specimens from the control gps showed remnants of hyalinized tissues with less organized PDL fibers at the compression site at day 21, so the process of necrotic tissues removal and the subsequent root resorption process were still active at this time.

So, it seems that, even if the hyalinization process occurred with the PBM, the process of hyalinization and removal of the hyalinized tissues did not take a long time with the PBM like without PBM. Thus, the result was overall less damage to the root structures. Furthermore, the better reorganization of the PDL collagen fibers, we found in the current study, aligns with the previous report by Maia et al. [61] who reported the positive role of PBM in promoting stromal cell proliferation, enhancing angiogenesis, and resulting in enhanced architectural reorganisation of periodontal collagen fibres.

Besides, Laser biomodulation might also speed up the inflammatory process and change it such that it is less severe and lasts for a shorter time. This might help protect the cementoblasts and pre-cementum layer better since they would not be faced with a lot of stress for a long time. This might lower the chance of root resorption and injury, despite the ongoing high level of bone resorption processes at the compression site [15]. 

Additionally, histologic and histomorphometric analyses indicated a higher quantity of dilated blood capillaries crowded with blood cells in the laser gps in comparison to the control gps. This finding corresponds with the studies conducted by Akin et al. [62] and Yoo et al. [63] which indicated that mitochondrial absorption of light photons elevates the concentrations of reactive oxygen species (ROS) and nitric oxide (NO). The NO might make the microvasculature more permeable and enhance the capillary vascularization. These observations, together with our results, corroborate the concept that PBM may reduce OIIRR by influencing the vasculature, since enhanced microvascular permeability may result in an expanded osteoclast numbers. Furthermore, the enhancement of tissue oxygenation may stimulate cellular signaling pathways, hence elevating transcription factors, like nuclear factor kappa B (NF-κB), which is critical for RANKL-induced differentiation of the osteoclasts [64, 65]. The rise in osteoclastic bone resorption activities causes a quick drop in compressive stresses, which decreases creating of hyalinized tissue [48]. Besides, Brezniak and Wasserstein [2] claimed that the OIIRR is part of getting rid of the dead tissues that happens when blood delivery to the PDL becomes inturrupted. So, we suspect that enhancing tissue vascularization might safeguard radical tissues more by avoiding hyalinization and tissue necrosis from the start.

Moreover, our histologic observations showed that a new layer of reparative cementum were clearly formed over nearly every pre-formed resorption lacunae in the laser gps at day 21. In contrast, rats in the control gps had a reduced prevalence and decreased thickness of newly made reparative cementum. This conclusion aligns with the results of Altan et al.,[20] that revealed the positive impacts of PBM in speeding and enhancing the healing and the repair of OIIRR. They said that PBM speeds up the primary release of the uncalcified pre-cementum layer by repair cells, fibroblast-like cells, and cementoblasts. subsequently, the PDL fibers reattach. This offers fast protection for the root tissues against the activity of resorbing cells.

Conversely, several investigations indicated discrepancies in their findings compared to the current research [16, 66]. They stated that laser irradiation did not significantly contribute to the prevention and reduction of the OIIRR. These contradictory outcomes could result from inadequate force magnitudes, varying laser settings, or an insufficient experimental duration.

A lot of researchers have said that root resorption became much worse as the force magnitudes increase [5, 46, 67]. However, some conflicting findings show that high forces did not cause root resorption to happen more [68, 69]. In the current study, we observed a rise in the amount of OIIRR in the high-force subgroups over the low-force subgroups for both the control and laser gps. However, this increase in the amount of root resorption between low- and high-force subgroups was statistically not significant (*p*>0.05). To be mentioned, our study was designed mainly to examine the effect of PBM on the OIIRR under two orthodontic force magnitudes that are not extremely high, rather than investigating the absolute relation between the force magnitude and root resorption. Although our results were consistent with previous reports [68, 70], it seems to be better to use more than two force magnitudes with a great difference between them for better investigation of the relationship between the force magnitudes and OIIRR.

In the current study, time-related root resorption was obviously seen in the control gps (high- and low-force). The amount of root resorption increased significantly (*p* < 0.001) from day 7 to day 21 for the control low-force gp (CL) and control high-force gp (CH). This inference is in conformity with the previous reports [4, 7]. 

On the other side, in the current study, a statistically significant change in the amount of root resorption from day 7 to day 21 was observed in the laser low-force gp (LL) (*p* = 0.958). While no statistically significant change in the amount of root resorption from day 7 to day 21 was found in laser high-force gp (LH) (*p* = 0.989). The PBM, did not only prevent the increase in amount of OIIRR with time, but also slightly decreased the amount of root resorption from day 7 to day 21 for both LL and LH gps, despite this decrease was statistically not significant. These results reflect the effect of PBM in preventing the progress of the OIIRR, in addition to its reparative effect which induces and accelerates the repair of the radicular tissues after being resorbed. Our histological observations, including the early removal of the hyalinized tissues, the increased deposition of the reparative cementum, the enhanced revascularization and the proper re-attachment and reorganization of the PDL fibers with PBM, also confirmed this hypothesis.

Regarding the limitations of the present study, we used a non-contact irradiation method for the PBM, the irradiating probe was kept about 1 mm from the target tissues. We tried to keep the distance, between the probe and tissues, constant in all the irradiation sessions, for all the animals, by contacting the tissues by the probe before active irradiation, followed by just de-contacting the tissue and then activating the laser beam irradiation. However, the accurate measurement of this distance during the irradiation sessions was difficult to be reached due to the narrow working field and difficult accessibility. Also, only cross-sections of the M1 were used for histological and histomorphometic analysis in the present study. Despite it has been proven that longitudinal sections in the mid-sagittal plane might not be an accurate representation of the entire spectrum of histological events[42, 70, 71], it seems to be better to use a combination of cross sections and longitudinal sections of the root for better evaluation of the resorption craters. However, this would consume more time, require larger sample size and increase the cost of the experiment. Efforts were done to take cross sections of almost whole the cervical two thirds of the root to detect most of the resorption craters and reach satisfying results. Besides, root resorption crater is a volumetric reaction and the histologic sections are still two-dimensional representation of this three-dimensional tissue reaction. Although we tried to take large number of sections to reach a realistic estimate of the assay tested, the volumetric representation of the tissue events could not be reached without three-dimensional evaluating methods like; scanning electron micrography and micro-computed scan. The last but not the least, the present study is a single-sex study which may have some limitations due to the fact that only male animals were studied. To the best of what we know, there is no clear information on what differences might be observed between the sexes following PBM treatment, and it’s considered as one of limitations of the current study. Finally, further prolonged studies, with larger sample size, prolonged time intervals and variable laser parameters, are recommended to determine the appropriate time of intervention with PBM during orthodontic tooth movement, which maximizes the reparative effect of PBM without increasing the OIIRR in any phase of orthodontic tooth movement.

## Conclusions

Within the limitations of the current study, we can conclude that application of both 10–15 g and 50 g of mesializing forces for 21 days can cause significant orthodontic-induced root resorption (OIIRR) of the maxillary first molars in Albino rats. In addition, photo-biomodulation therapy (PBM) applied during exertion of light- (10–15 g) and heavy- (50 g) orthodontic loads can significantly reduce the total amount of root resorption and enhance/accelerate the healing of OIIRR. However, the effect of PBM on OIIRR is time-related effect. Finally, PBM has significant reparative effects on OIIRR, under both light- and heavy- orthodontic force magnitudes, including enhancement of the regeneration and reorganization of PDL fibers, improvement of the PDL vasculature and stimulation of angiogenesis, in addition to stimulation of reparative cementum deposition.

## Supplementary Information

Supplementary Material 1.

Supplementary Material 2.

## Data Availability

All data included in this current study are available from the corresponding author upon request.

## Abbreviations

PBM: Photo-biomodulation therapy
OIIRR: Orthodontic-induced inflammatory root resorption
OTM: Orthodontic tooth movement
M1: Maxillary left first molar
BVS: Blood vessels
gp: group
PDL: Periodontal ligament
OPG: Osteoprotegerin
RANK: Receptor activator of nuclear factor kappa-B receptor
RANKL: Receptor activator of nuclear factor kappa-B ligand
TRAP: Tartrate-resistant acid phosphatase
ROS: Reactive oxygen species
NO: Nitric oxide
DB: Distal-buccal

## References

[1] Gul Amuk N, Kurt G, Guray E. Effects of photobiomodulation and ultrasound applications on orthodontically induced inflammatory root Resorption; transcriptional alterations in OPG, RANKL, Cox-2: an experimental study in rats. Photomed Laser Surg. 2018;36:653–9. 31697637 10.1089/pho.2018.4508

[2] Brezniak N, Wasserstein A. Orthodontically induced inflammatory root resorption. Part I: the basic science aspects. Angle Orthod. 2002;72:175–9. 11999941 10.1043/0003-3219(2002)072<0175:OIIRRP>2.0.CO;2

[3] Abass SK, Hartsfield JK. Investigation of Genetic Factors Affecting Complex Traits Using External Apical Root Resorption as a Model. Semin Orthod. 2008;14:115-24.

[4] Nanekrungsan K, Patanaporn V, Janhom A, Korwanich N. External apical root resorption in maxillary incisors in orthodontic patients: associated factors and radiographic evaluation. Imaging Sci Dent. 2012;42:147–54. 23071964 10.5624/isd.2012.42.3.147 PMC3465756

[5] Currell S, Liaw A, Grant P, Esterman A, Nimmo A. Orthodontic mechanotherapies and their influence on external root resorption: A systematic review. Am J Orthod Dentofac Orthop. 2019;155:313–29. 10.1016/j.ajodo.2018.10.015 30826034

[6] Jung YH, Cho BH. External root resorption after orthodontic treatment: a study of contributing factors. Imaging Sci Dent. 2011;41:17–21. 21977469 10.5624/isd.2011.41.1.17 PMC3174460

[7] Segal GR, Schiffman PH, Tuncay OC. Meta analysis of the treatment-related factors of external apical root resorption. Orthod Craniofac Res. 2004;7:71–8. 15180086 10.1111/j.1601-6343.2004.00286.x

[8] Yassir YA, McIntyre GT, Bearn DR. Orthodontic treatment and root resorption: an overview of systematic reviews. Eur J Orthod. 2020;43:442–56. 10.1093/ejo/cjaa058 33215186

[9] Proffit WR, Fields HW Jr, Sarver DM. Contemporary orthodontics. 4th ed. ed. ST Louis: CV Mosby: Elsevier Health Sciences;; 2006.

[10] Seifi M, Atri F, Yazdani MM. Effects of low-level laser therapy on orthodontic tooth movement and root resorption after artificial socket preservation. Dent Res J (Isfahan). 2014;11:61–6. 24688562 PMC3955317

[11] Anders JJ, Arany PR, Baxter GD, Lanzafame RJ. Light-Emitting diode therapy and Low-Level light therapy are photobiomodulation therapy. Photobiomodul Photomed Laser Surg. 2019;37:63–5. 31050924 10.1089/photob.2018.4600

[12] Sobouti F, Khatami M, Chiniforush N, Rakhshan V, Shariati M. Effect of single-dose low-level helium-neon laser irradiation on orthodontic pain: a split-mouth single-blind placebo-controlled randomized clinical trial. Prog Orthod. 2015;16:32. 26446930 10.1186/s40510-015-0102-0 PMC4883614

[13] Altan BA, Sokucu O, Ozkut MM, Inan S. Metrical and histological investigation of the effects of low-level laser therapy on orthodontic tooth movement. Lasers Med Sci. 2012;27:131–40. 21038101 10.1007/s10103-010-0853-2

[14] Heravi F, Moradi A, Ahrari F. The effect of low level laser therapy on the rate of tooth movement and pain perception during canine Retraction. Oral Health Dent Manag. 2014;13:183–8. 24984620

[15] Suzuki SS, Garcez AS, Suzuki H, Ervolino E, Moon W, Ribeiro MS. Low-level laser therapy stimulates bone metabolism and inhibits root resorption during tooth movement in a rodent model. J Biophotonics. 2016;9:1222–35. 27647761 10.1002/jbio.201600016

[16] Vasconcelos EC, Henriques JF, Sousa MV, et al. Low-Level laser action on orthodontically induced root resorption: histological and histomorphometric evaluation. J Lasers Med Sci. 2016;7:146–51. 28144433 10.15171/jlms.2016.25 PMC5262479

[17] Vilela RG, Gjerde K, Frigo L, et al. Histomorphometric analysis of inflammatory response and necrosis in re-implanted central incisor of rats treated with low-level laser therapy. Lasers Med Sci. 2012;27:551–7. 21617972 10.1007/s10103-011-0937-7 PMC3319885

[18] Nayyer N, Tripathi T, Ganesh G, Rai P. Impact of photobiomodulation on external root resorption during orthodontic tooth movement in humans – A systematic review and meta-analysis. J Oral Biology Craniofac Res. 2022;12:469–80. 10.1016/j.jobcr.2022.05.014 PMC9178480 35692967

[19] Charan J, Biswas T. How to calculate sample size for different study designs in medical research? Indian J Psychol Med. 2013;35:121–6. 24049221 10.4103/0253-7176.116232 PMC3775042

[20] Altan AB, Bicakci AA, Mutaf HI, Ozkut M, Inan VS. The effects of low-level laser therapy on orthodontically induced root resorption. Lasers Med Sci. 2015;30:2067–76. 25633918 10.1007/s10103-015-1717-6

[21] Faul F, Erdfelder E, Lang A-G, Buchner A. G*Power 3: A flexible statistical power analysis program for the social, behavioral, and biomedical sciences. Behav Res Methods. 2007;39:175–91. 17695343 10.3758/bf03193146

[22] Suresh K. An overview of randomization techniques: an unbiased assessment of outcome in clinical research. J Hum Reprod Sci. 2011;4:8–11. 21772732 10.4103/0974-1208.82352 PMC3136079

[23] Prism G. version 5.01. *GraphPad Software Inc.: San Diego, CA, USA.* 2007.

[24] Muhlhausler BS, Bloomfield FH, Gillman MW. Whole animal experiments should be more like human randomized controlled trials. PLoS Biol. 2013;11:e1001481. 23424284 10.1371/journal.pbio.1001481 PMC3570551

[25] Guan L, Lin S, Yan W, Chen L, Wang X. Effects of calcitonin on orthodontic tooth movement and associated root resorption in rats. Acta Odontol Scand. 2017;75:595–602. 28814141 10.1080/00016357.2017.1365375

[26] Franzen TJ, Zahra SE, El-Kadi A, Vandevska-Radunovic V. The influence of low-level laser on orthodontic relapse in rats. Eur J Orthod. 2015;37:111–7. 25287057 10.1093/ejo/cju053

[27] Yassin A, Shehata F, Al-Sawa A, Karam S. Effect of Low-Level laser therapy on orthodontic induced inflamatory root resorption in rats. Alexandria Dent J. 2020;45:62–7.

[28] Olfert ED, Cross BM, McWilliam AA. Guide to the care and use of experimental animals. Vol 1: Canadian Council on Animal Care Ottawa; 1993:298.

[29] Eriksen EF, Axelrod DW, Melsen F. Bone histomorphometry. New York, USA: Raven Press; 1994:35-47.

[30] Kumar G. Orban’s oral histology & embryology. Volume 13. India: Elsevier Health Sciences; 2014. pp. 431–7.

[31] Schneider CA, Rasband WS, Eliceiri KW. NIH image to imageJ: 25 years of image analysis. Nat Methods. 2012;9:671–5. 22930834 10.1038/nmeth.2089 PMC5554542

[32] Fracalossi ACC, Santamaria M Jr, Consolaro MFM-O, Consolaro A. Experimental tooth movement in murines: study period and direction of microscopic sections. Dent Press J Orthod. 2009;14:143–57.

[33] Watarai H, Warita H, Soma K. Effect of nitric oxide on the recovery of the hypofunctional periodontal ligament. J Dent Res. 2004;83:338–42. 15044510 10.1177/154405910408300413

[34] Kirkpatrick LA, Feeney BC. A Simple Guide to IBM SPSS: for Version 23.0. 13th ed. New York: Delmar Learning; 2015.

[35] Erratum to. A Guideline of Selecting and Reporting Intraclass Correlation Coefficients for Reliability Research [J Chiropr Med 2016;15(2):155–163]. *J Chiropr Med.* 2017;16:346. 10.1016/j.jcm.2016.02.012 PMC4913118 27330520

[36] Hikida T, Yamaguchi M, Shimizu M, Kikuta J, Yoshino T, Kasai K. Comparisons of orthodontic root resorption under heavy and jiggling reciprocating forces during experimental tooth movement in a rat model. Korean J Orthod. 2016;46:228–41. 27478800 10.4041/kjod.2016.46.4.228 PMC4965594

[37] Cambois E, Duthé G, Meslé F. Global Trends in Life Expectancy and Healthy Life Expectancy: Oxford University Press; 2023.

[38] Xu X, Zhou J, Yang F, Wei S, Dai H. Using Micro-Computed tomography to evaluate the dynamics of orthodontically induced root resorption repair in a rat model. PLoS ONE. 2016;11:e0150135. 26930605 10.1371/journal.pone.0150135 PMC4773112

[39] Hellsing E, Hammarstrom L. The hyaline zone and associated root surface changes in experimental orthodontics in rats: a light and scanning electron microscope study. Eur J Orthod. 1996;18:11–8. 8746173 10.1093/ejo/18.1.11

[40] Jager A, Kunert D, Friesen T, Zhang D, Lossdorfer S, Gotz W. Cellular and extracellular factors in early root resorption repair in the rat. Eur J Orthod. 2008;30:336–45. 18632841 10.1093/ejo/cjn012

[41] Proffit W, Fields H, Sarver D. The biological basis of orthodontic therapy. *Contemporary Orthodontics. 3ed. St. Louis: Mosby.* 2000:296–317.

[42] van Leeuwen EJ, Maltha JC, Kuijpers-Jagtman AM. Tooth movement with light continuous and discontinuous forces in beagle dogs. Eur J Oral Sci. 1999;107:468–74. 10625106 10.1046/j.0909-8836.1999.eos107608.x

[43] Yokoya K, Sasaki T, Shibasaki Y. Distributional changes of osteoclasts and pre-osteoclastic cells in periodontal tissues during experimental tooth movement as revealed by quantitative immunohistochemistry of H+-ATPase. J Dent Res. 1997;76:580–7. 9042081 10.1177/00220345970760010901

[44] Choi J, Baek SH, Lee JI, Chang YI. Effects of clodronate on early alveolar bone remodeling and root resorption related to orthodontic forces: a histomorphometric analysis. *Am J Orthod Dentofacial Orthop.* 2010;138:548 e541-548; discussion 548–549. 10.1016/j.ajodo.2010.01.031 21055592

[45] Gonzales C, Hotokezaka H, Karadeniz EI, et al. Effects of fluoride intake on orthodontic tooth movement and orthodontically induced root resorption. Am J Orthod Dentofac Orthop. 2011;139:196–205. 10.1016/j.ajodo.2009.05.029 21300248

[46] Gonzales C, Hotokezaka H, Yoshimatsu M, Yozgatian JH, Darendeliler MA, Yoshida N. Force magnitude and duration effects on amount of tooth movement and root resorption in the rat molar. Angle Orthod. 2008;78:502–9. 18416627 10.2319/052007-240.1

[47] Kohno T, Matsumoto Y, Kanno Z, Warita H, Soma K. Experimental tooth movement under light orthodontic forces: rates of tooth movement and changes of the periodontium. J Orthod. 2002;29:129–35. 12114463 10.1093/ortho/29.2.129

[48] Suzuki SS, Garcez AS, Reese PO, Suzuki H, Ribeiro MS, Moon W. Effects of corticopuncture (CP) and low-level laser therapy (LLLT) on the rate of tooth movement and root resorption in rats using micro-CT evaluation. Lasers Med Sci. 2018;33:811–21. 29282560 10.1007/s10103-017-2421-5

[49] Ren Y, Maltha JC, Kuijpers-Jagtman AM. The rat as a model for orthodontic tooth movement—a critical review and a proposed solution. Eur J Orthod. 2004;26:483–90. 15536836 10.1093/ejo/26.5.483

[50] Chung H, Dai T, Sharma SK, Huang YY, Carroll JD, Hamblin MR. The nuts and bolts of low-level laser (light) therapy. Ann Biomed Eng. 2012;40:516–33. 22045511 10.1007/s10439-011-0454-7 PMC3288797

[51] Qadri T, Miranda L, Tuner J, Gustafsson A. The short-term effects of low‐level lasers as adjunct therapy in the treatment of periodontal inflammation. J Clin Periodontol. 2005;32:714–9. 15966876 10.1111/j.1600-051X.2005.00749.x

[52] Coluzzi DJ. Fundamentals of dental lasers: science and instruments. Dent Clin North Am. 2004;48:751–70. 15464551 10.1016/j.cden.2004.05.003

[53] Ekizer A, Uysal T, Guray E, Akkus D. Effect of LED-mediated-photobiomodulation therapy on orthodontic tooth movement and root resorption in rats. Lasers Med Sci. 2015;30:779–85. 23990217 10.1007/s10103-013-1405-3

[54] Hamblin MR, Demidova TN. Mechanisms of low level light therapy. Mechanisms for low-light therapy. Proc. of SPIE. Vol 6140; 2006:614001.

[55] Hillenkamp F. Interaction between Laser Radiation and Biological Systems. In: Hillenkamp F, Pratesi R, Sacchi CA, eds. Lasers in Biology and Medicine. Boston, MA: Springer US; 1980:37-68.

[56] Youssef M, Ashkar S, Hamade E, Gutknecht N, Lampert F, Mir M. The effect of low-level laser therapy during orthodontic movement: a preliminary study. Lasers Med Sci. 2008;23:27–33. 17361391 10.1007/s10103-007-0449-7

[57] Cruz DR, Kohara EK, Ribeiro MS, Wetter NU. Effects of low-intensity laser therapy on the orthodontic movement velocity of human teeth: a preliminary study. Lasers Surg Med. 2004;35:117–20. 15334614 10.1002/lsm.20076

[58] Goulart CS, Nouer PR, Mouramartins L, Garbin IU. De Fatima Zanirato Lizarelli R. Photoradiation and orthodontic movement: experimental study with canines. Photomed Laser Surg. 2006;24:192–6. 16706698 10.1089/pho.2006.24.192

[59] Passarella S, Karu T. Absorption of monochromatic and narrow band radiation in the visible and near IR by both mitochondrial and non-mitochondrial photoacceptors results in photobiomodulation. J Photochem Photobiol B. 2014;140:344–58. 25226343 10.1016/j.jphotobiol.2014.07.021

[60] Genc G, Kocadereli I, Tasar F, Kilinc K, El S, Sarkarati B. Effect of low-level laser therapy (LLLT) on orthodontic tooth movement. Lasers Med Sci. 2013;28:41–7. 22350425 10.1007/s10103-012-1059-6

[61] Maia LG, Alves AV, Bastos TS, et al. Histological analysis of the periodontal ligament and alveolar bone during dental movement in diabetic rats subjected to low-level laser therapy. J Photochem Photobiol B. 2014;135:65–74. 24814932 10.1016/j.jphotobiol.2014.03.023

[62] Akin E, Gurton AU, Olmez H. Effects of nitric oxide in orthodontic tooth movement in rats. Am J Orthod Dentofac Orthop. 2004;126:608–14. 10.1016/S0889540604004494 15520694

[63] Yoo SK, Warita H, Soma K. Duration of orthodontic force affecting initial response of nitric oxide synthase in rat periodontal ligaments. J Med Dent Sci. 2004;51:83–8. 15137469

[64] Chen AC, Arany PR, Huang YY, et al. Low-level laser therapy activates NF-kB via generation of reactive oxygen species in mouse embryonic fibroblasts. PLoS ONE. 2011;6:e22453. 21814580 10.1371/journal.pone.0022453 PMC3141042

[65] Yu M, Qi X, Moreno JL, Farber DL, Keegan AD. NF-κB signaling participates in both RANKL-and IL-4–induced macrophage fusion: receptor cross talk leads to alterations in NF-κB pathways. J Immunol 2011:1002628. 10.4049/jimmunol.1002628 PMC3150418 21734075

[66] Khaw CMA, Dalci O, Foley M, Petocz P, Darendeliler MA, Papadopoulou AK. Physical properties of root cementum: part 27. Effect of low-level laser therapy on the repair of orthodontically induced inflammatory root resorption: A double-blind, split-mouth, randomized controlled clinical trial. Am J Orthod Dentofac Orthop. 2018;154:326–36. 10.1016/j.ajodo.2018.04.022 30173835

[67] Chan E, Darendeliler MA. Physical properties of root cementum: part 7. Extent of root resorption under areas of compression and tension. Am J Orthod Dentofac Orthop. 2006;129:504–10. 10.1016/j.ajodo.2004.12.018 16627176

[68] Owman-Moll P, Kurol J, Lundgren D. The effects of a four-fold increased orthodontic force magnitude on tooth movement and root resorptions. An intra-individual study in adolescents. Eur J Orthod. 1996;18:287–94. 8791892 10.1093/ejo/18.3.287

[69] Owman-Moll P. Orthodontic tooth movement and root resorption with special reference to force magnitude and duration. A clinical and histological investigation in adolescents. Swed Dent J Suppl. 1995;105:1–45. 7638765

[70] Owman-Moll P, Kurol J, Lundgren D. Effects of a doubled orthodontic force magnitude on tooth movement and root resorptions. An inter-individual study in adolescents. Eur J Orthod. 1996;18:141–50. 8670926 10.1093/ejo/18.2.141

[71] von Böhl M, Kuijpers-Jagtman AM. Hyalinization during orthodontic tooth movement: a systematic review on tissue reactions. Eur J Orthod. 2009;31:30-36. 10.1093/ejo/cjn080 19073957

