# Supplementary material for: Effect of photobiomodulation therapy on orthodontic-induced inflammatory root resorption in male albino rats
Source: BMC Oral Health. 2025 Dec 11;26:99. doi: 10.1186/s12903-025-07217-2 (PMC12801967; doi:10.1186/s12903-025-07217-2)
Supplement: Supplementary file 1 — Supplementary Material 1. [file 12903_2025_7217_MOESM1_ESM.docx]

**Table (I): Test of Normality check using Shapiro-Wilk test for different parameters (BVS)**

|  | **Kolmogorov-Smirnov** | | | **Shapiro-Wilk** | | |
| --- | --- | --- | --- | --- | --- | --- |
|  | **Statistic** | **df** | **Sig.** | **Statistic** | **df** | **Sig.** |
| **Control Low (7)** | 0.152 | 8 | 0.200 | 0.965 | 8 | 0.857 |
| **Control Low (21)** | 0.300 | 8 | 0.032^*^ | 0.872 | 8 | 0.156 |
| **Control High (7)** | 0.216 | 8 | 0.200 | 0.906 | 8 | 0.324 |
| **Control High (21)** | 0.240 | 8 | 0.195 | 0.858 | 8 | 0.114 |
| **Laser Low (7)** | 0.228 | 8 | 0.200 | 0.945 | 8 | 0.658 |
| **Laser Low (21)** | 0.160 | 8 | 0.200 | 0.937 | 8 | 0.585 |
| **Laser High (7)** | 0.358 | 8 | 0.003^*^ | 0.773 | 8 | 0.015^*^ |
| **Laser High (21)** | 0.335 | 8 | 0.009^*^ | 0.669 | 8 | 0.001^*^ |

*: Statistically significant at p ≤ 0.05

**Table (II): Test of Normality check using Shapiro-Wilk test for different parameters (root resorption)**

|  | **Kolmogorov-Smirnov** | | | **Shapiro-Wilk** | | |
| --- | --- | --- | --- | --- | --- | --- |
|  | **Statistic** | **df** | **Sig.** | **Statistic** | **df** | **Sig.** |
| **Control Low (7)** | 0.201 | 8 | 0.200 | 0.897 | 8 | 0.272 |
| **Control Low (21)** | 0.213 | 8 | 0.200 | 0.858 | 8 | 0.114 |
| **Control High (7)** | 0.215 | 8 | 0.200 | 0.892 | 8 | 0.242 |
| **Control High (21)** | 0.189 | 8 | 0.200 | 0.916 | 8 | 0.395 |
| **Laser Low (7)** | 0.189 | 8 | 0.200 | 0.859 | 8 | 0.117 |
| **Laser Low (21)** | 0.155 | 8 | 0.200 | 0.919 | 8 | 0.423 |
| **Laser High (7)** | 0.210 | 8 | 0.200 | 0.881 | 8 | 0.191 |
| **Laser High (21)** | 0.183 | 8 | 0.200 | 0.901 | 8 | 0.294 |

*: Statistically significant at p ≤ 0.05
