# Supplementary material for: Effect of photobiomodulation therapy on orthodontic-induced inflammatory root resorption in male albino rats
Source: BMC Oral Health. 2025 Dec 11;26:99. doi: 10.1186/s12903-025-07217-2 (PMC12801967; doi:10.1186/s12903-025-07217-2)

The Bland-Altman test for repeated measurements

Table I : Agreement between root resorption readings and BVs count using Bland-Altman test

|  | Root Resorption | BVs |
| --- | --- | --- |
| Mean difference | -0.01 | 0.10 |
| 95% CI of mean difference | -0.12, 0.09 | -0.43 to 0.63 |
| P value (H0: Mean = 0) | 0.795 | 0.678 |
| Lower limit | -0.31 | -1.35 |
| 95% CI of lower limit | -0.50 to -0.12 | -2.28 to -0.41 |
| Upper limit | 0.28 | 1.55 |
| 95% CI of upper limit | 0.09 to 0.47 | 0.61 to 2.48 |


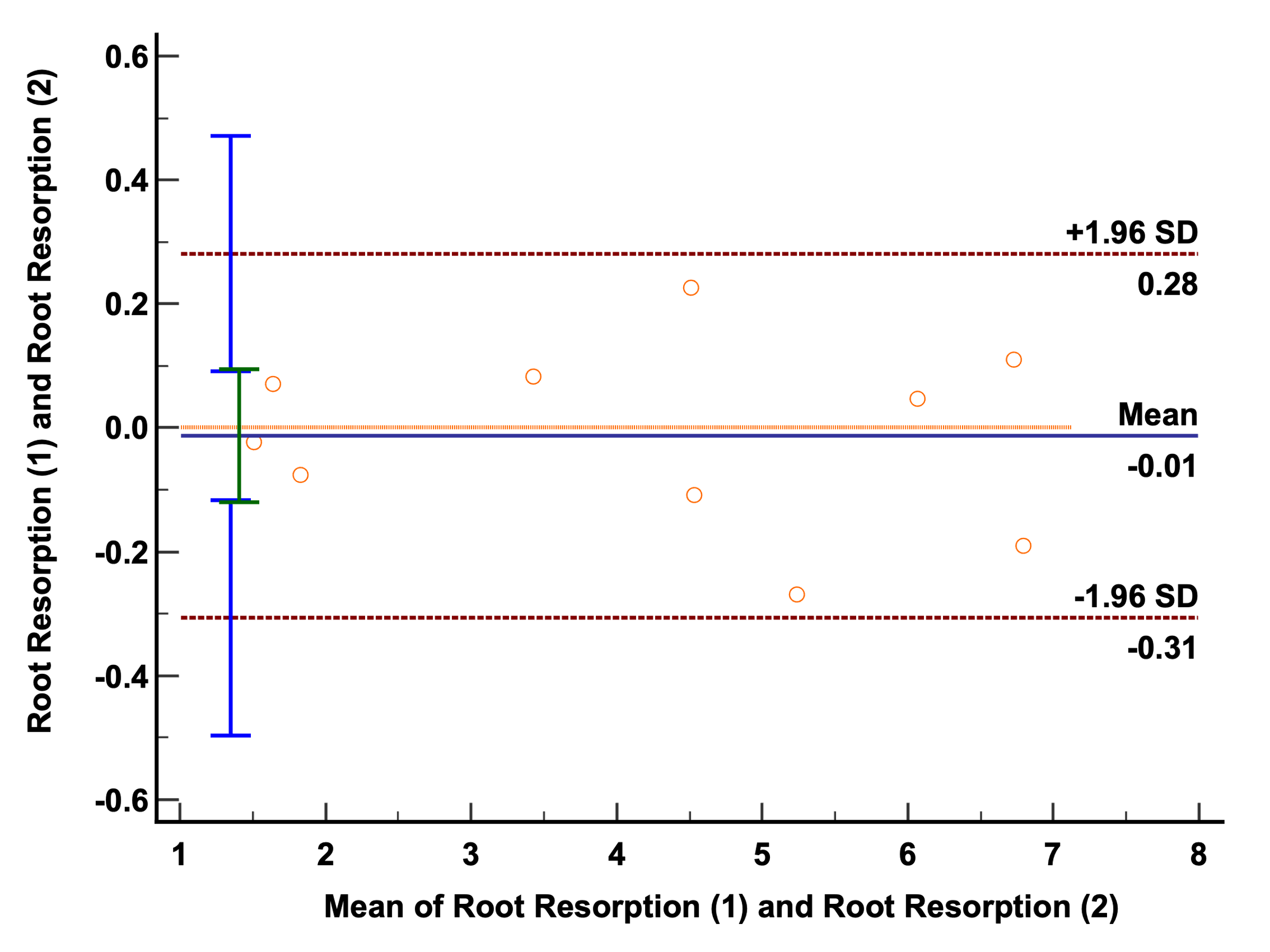


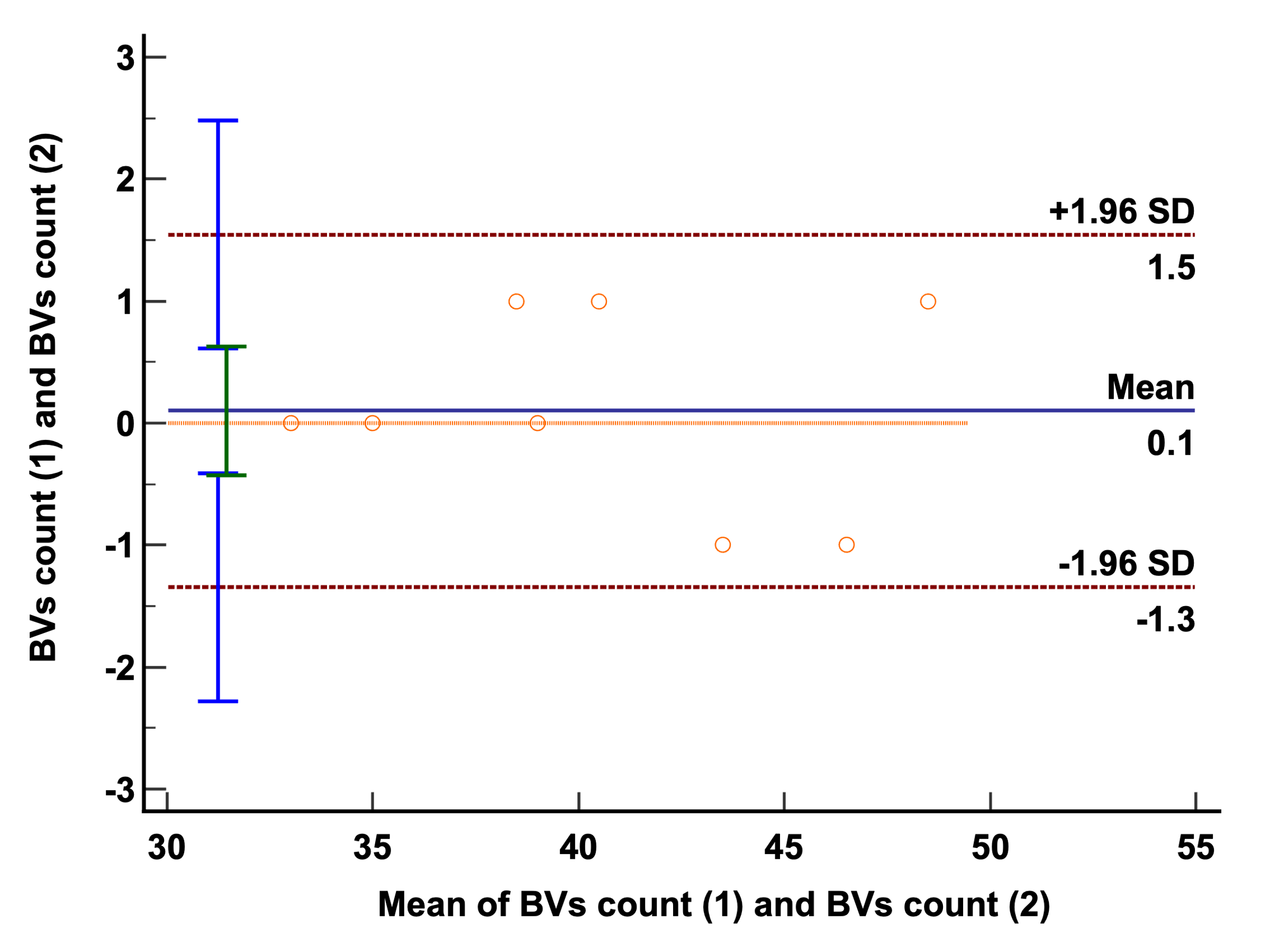

Supplement: Supplementary file 2 — Supplementary Material 2. [file 12903_2025_7217_MOESM2_ESM.docx]
